# Anti‐PD‐1 treatment protects against seizure by suppressing sodium channel function

**DOI:** 10.1111/cns.14504

**Published:** 2023-10-30

**Authors:** Yuling Yang, Zhiyun Chen, Jing Zhou, Shize Jiang, Guoxiang Wang, Li Wan, Jiangning Yu, Min Jiang, Yulong Wang, Jie Hu, Xu Liu, Yun Wang

**Affiliations:** ^1^ Department of Neurology, Institutes of Brain Science, State Key Laboratory of Medical Neurobiology and MOE Frontiers Center for Brain Science, Zhongshan Hospital Fudan University Shanghai China; ^2^ Rehabilitation Center Shenzhen Second People's Hospital/The First Affiliated Hospital of Shenzhen University Health Science Center Shenzhen China; ^3^ Department of Neurosurgery, Huashan Hospital Fudan University Shanghai China

**Keywords:** epilepsy, Nav1.6, PD‐1 receptor, sodium channel

## Abstract

**Aims:**

Although programmed cell death protein 1 (PD‐1) typically serves as a target for immunotherapies, a few recent studies have found that PD‐1 is expressed in the nervous system and that neuronal PD‐1 might play a crucial role in regulating neuronal excitability. However, whether brain‐localized PD‐1 is involved in seizures and epileptogenesis is still unknown and worthy of in‐depth exploration.

**Methods:**

The existence of PD‐1 in human neurons was confirmed by immunohistochemistry, and PD‐1 expression levels were measured by real‐time quantitative PCR (RT‐qPCR) and western blotting. Chemoconvulsants, pentylenetetrazol (PTZ) and cyclothiazide (CTZ), were applied for the establishment of in vivo (rodents) and in vitro (primary hippocampal neurons) models of seizure, respectively. SHR‐1210 (a PD‐1 monoclonal antibody) and sodium stibogluconate (SSG, a validated inhibitor of SH2‐containing protein tyrosine phosphatase‐1 [SHP‐1]) were administrated to investigate the impact of PD‐1 pathway blockade on epileptic behaviors of rodents and epileptiform discharges of neurons. A miRNA strategy was applied to determine the impact of PD‐1 knockdown on neuronal excitability. The electrical activities and sodium channel function of neurons were determined by whole‐cell patch‐clamp recordings. The interaction between PD‐1 and α‐6 subunit of human voltage‐gated sodium channel (Nav1.6) was validated by performing co‐immunostaining and co‐immunoprecipitation (co‐IP) experiments.

**Results:**

Our results reveal that PD‐1 protein and mRNA levels were upregulated in lesion cores compared with perifocal tissues of surgically resected specimens from patients with intractable epilepsy. Furthermore, we show that anti‐PD‐1 treatment has anti‐seizure effects both in vivo and in vitro. Then, we reveal that PD‐1 blockade can alter the electrophysiological properties of sodium channels. Moreover, we reveal that PD‐1 acts together with downstream SHP‐1 to regulate sodium channel function and hence neuronal excitability. Further investigation suggests that there is a direct interaction between neuronal PD‐1 and Nav1.6.

**Conclusion:**

Our study reveals that neuronal PD‐1 plays an important role in epilepsy and that anti‐PD‐1 treatment protects against seizures by suppressing sodium channel function, identifying anti‐PD‐1 treatment as a novel therapeutic strategy for epilepsy.

## Graphical abstract

The blockade of PD‐1 alleviates neuronal overexcitation by reinstating sodium channel inactivation, indicating anti‐PD‐1 treatment as a novel therapeutic strategy for epilepsy.

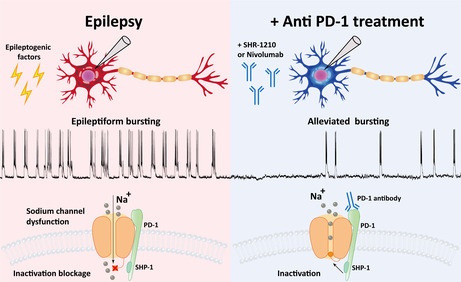

## 1 INTRODUCTION

Programmed cell death protein 1 (PD‐1), as an immune checkpoint, has recently attracted considerable attention for its role in tumor immunotherapy.^1^, ^2^, ^3^ PD‐1 is well known as a coinhibitory receptor expressed on the cell surface of T cells. The PD‐1 ligands, programmed cell death 1 ligand 1 (PD‐L1) and ligand 2 (PD‐L2), signal through PD‐1 to contribute to immune homeostasis by regulating initial T‐cell activation, fine‐tuning T‐cell functions, and inhibiting the immune response; thus, PD‐1 plays a vital role in maintaining peripheral immune tolerance.^4^, ^5^ Regarding the role of PD‐1 and PD‐L1 in neurological diseases, several studies have found that PD‐L1 on glioblastoma and glioma can suppress antitumor immunity, thus facilitating the development of tumors.^6^, ^7^, ^8^, ^9^ Baruch et al., also found that the PD‐1 pathway may be targeted for the treatment of Alzheimer's disease.^10^, ^11^

It is becoming evident that many key immune signaling molecules are also expressed in neurons, and subsets of these molecules are now known to perform critical functions in the brain.^12^ Previous research has found that PD‐1 and its ligands are expressed in both the peripheral nervous system and central nervous system (CNS), such as in retinal ganglion cells and cerebral, hippocampal, thalamic, and dorsal root ganglion (DRG) neurons.^13^, ^14^, ^15^ However, only within the past few years has the role of neuronal PD‐1 been elucidated through research on the relationship between PD‐1 and pain.^15^, ^16^, ^17^ Nonetheless, there is a paucity of studies investigating the role of neuronal PD‐1 in CNS diseases, especially epilepsy. It is generally accepted that seizures are generated by abnormal, spontaneous, and synchronized neuronal firing,^18^ in which aberrant ion channel expression and function play an important role.^19^, ^20^ Voltage‐gated sodium channels (VGSCs) are responsible for the initiation and propagation of action potentials, and it is believed that modulating the expression and function of these channels can protect against certain types of seizures.^21^, ^22^ Nav1.6 is one of the main VGSCs in the adult brain, and it plays a role in the generation of persistent and resurgent currents, which makes it a critical determinant of neuronal excitability.^23^, ^24^ Resurgent sodium currents mediated by Nav1.6 may be important contributors to burst firing.^25^ There is sufficient evidence from both clinical research and experimental research supporting a potential role for Nav1.6 in facilitating epileptic encephalopathy.^26^, ^27^

Recently, researchers found that the activation of PD‐1 by PD‐L1 inhibited action potential induction and suppressed transient sodium currents in DRG neurons.^15^ The effect of the PD‐1 pathway on the function of sodium channels, in addition to the fact that PD‐1 is expressed on neurons, prompted us to investigate the contribution of PD‐1 to epilepsy and its potential as a therapeutic target. This study aimed to unravel some of the mysteries surrounding the association between PD‐1 and epilepsy by investigating the interactions between PD‐1 and sodium channels, particularly Nav1.6. Our findings indicated that the interaction between neuronal PD‐1 and Nav1.6 might play a crucial role in the generation of epilepsy. Moreover, we uncovered that blockade of PD‐1 could modulate neuronal excitability in neurons, at least partially by altering the electrophysiological properties of sodium channels. This study identified anti‐PD‐1 treatment as a novel therapeutic strategy for epilepsy.

## 2 MATERIALS AND METHODS

### 2.1 Subjects and tissue processing

Brain tissue samples from nine patients who had undergone neurosurgery for refractory epilepsy were obtained from Hua Shan Hospital (Fudan University in Shanghai, China). All procedures and experiments in this study were approved by the Ethics Committee of Fudan University (Ethics No. KY2015‐159). Informed consent was obtained from each patient for the use of brain tissue and access to medical records for research purposes. We collected core lesions and perilesional tissues from these  patients simultaneously for comparison using a standard procedure. Briefly, the origin of seizure and core lesion was determined by preoperative seizure monitoring, imaging, and electroencephalographic assessment. Patients were asked to discontinue the administration of anti‐seizure medicines for at least three seizures to monitor the origin of seizures through video electroencephalography or stereoelectroencephalography. Simultaneously, PET/MR scans were conducted on the patients and a comprehensive determination of the core area was thus made in combination with the results from EEG monitoring. For patients with a clearly identified origin who were suitable for epilepsy surgery, three‐dimensional reconstruction was conducted to plan the surgical scope of the lesion. During the surgery, navigation systems were utilized to achieve the resection of the lesion area. The scope of surgical resection will extend 1–2 cm beyond the nearest electrodes where no discharges were detected. The tissue located beyond the electrodes where no discharges were detected was defined as the perifocal tissue. Specimens were immediately stored in liquid nitrogen and maintained at −80°C (for western blotting (WB) and RT‐qPCR) or fixed in 4% Paraformaldehyde fixative (for immunohistochemistry). Patient information is listed in Table S1.

### 2.2 Reagents and plasmid

Reagents of PTZ, CTZ, and SHP‐1 inhibitor SSG were purchased from Sigma, USA. SHR‐1210, the blocker of PD‐1was a kind present sent by Shanghai Hengrui Pharmaceutical CO., LTD. Nivolumab was purchased from BMS, USA. The approximately 60 bp of the pre‐miRNA targeting PD‐1 mRNA sequence and non‐effective scrambled pre‐miRNA construct in a phSyn‐EGFP‐Mir155‐SV40‐puromycin vector were used to acutely silence PD‐1 expression and synthesized by Genechem Co., Ltd. (Shanghai, China).

PD‐1 pre‐miRNA (5′‐3′): TGAAGGTGGCATTTGCTCCCTGTTTTGGCCACTGACTGACAGGGAGCATGCCACCTTCACAGG.

Scrambled pre‐miRNA (5′‐3′): ACGTGACACGTTCGGAGAAGTTTTGGCCACTGACTGACTTCTCCGAGTGTCACGTCAGG.

### 2.3 Animals

Behavioral studies and electroencephalographic (EEG) recording were performed on male C57BL/6 mice weighing 22–26 g and male SD rats weighing 180–220 g. These animals were obtained from the Shanghai Experimental Animal Center of the Chinese Academy of Sciences. All the animals were maintained in an air‐conditioned room with a suitable temperature at 22 ± 1°C under a 12 h light/dark cycle (7:00 a.m. to 7:00 p.m., lights on), and food and water were available ad libitum. All the experimental procedures were approved by the Committee of Animal Use for Research and Education of Fudan University (Ethics No. Y2019‐053) and carried out in accordance with the Chinese National Science Foundation Animal Research Regulations. In the epileptic behavior experiments, we made all our efforts to minimize the number of animals used and their suffering, in accordance with the ethical guidelines for animal research.

### 2.4 Animal surgery procedure

Lateral ventricle cannula implantation: The animals were anesthetized with isoflurane and mounted in a stereotaxic apparatus with body temperature maintained at 37°C. The scalp was cut, and the skull was exposed. A little bur hole was made, with the tip of the guide cannula (26‐gauge, 2.0 mm) placed above the left lateral ventricle in mice (AP −0.5 mm, ML −1.0 mm, DP −2.0 mm). Similarly, the guide cannula (22‐gauge, 4 mm) for rat was placed above the left lateral ventricle in rat (AP −0.3 mm, ML −1.03 mm, DP −4.0 mm). The guide cannula was affixed to the skull with dental acrylic. The injection cannula was 30 gauge, 2.2 mm for mice and 27 gauge, 5.0 mm for rat.

Electrode implantation surgery: Two bur holes were drilled with two little stainless‐steel screws (2‐mm diameter and 2‐mm length) embedded, one serving as a recording electrode in the right skull above the hippocampus of mice (AP −1.8 mm and ML 1.4 mm) or rats (AP −3.8 mm and ML 2.0 mm) and the other as reference electrode above the forehead of mice (AP 1.5 mm and ML −1.5 mm) or rats (AP 2.5 mm and ML −2.5 mm). Two screws were then connected to a connector plug with wires for later connecting to recording leads. Then, screws and connector plug were affixed to the skull with dental acrylic. After surgery, animals were kept for recovery for at least 5 days before the experiments.

### 2.5 Immunofluorescence

Fixed brain tissues were gradiently dehydrated using 15% and 30% sucrose in PBS, embedded in OCT, and frozen at −80°C before sectioning. A total of 40‐μm thick slides were sectioned using a freezing microtome and then blocked in a blocking buffer consisting of 4% donkey serum and 0.25% Triton X‐100 for 2 h at room temperature. Floating slides were incubated overnight at 4°C in primary antibodies Guinea Pig anti‐NeuN (Millipore, ABN90P, 1:800), Mouse anti‐PD‐1 (Proteintech, 66220‐1‐Ig, 1:500), and Rabbit anti‐Nav1.6 (Alomone Labs, ASC‐009, 1:200) diluted in blocking buffer. Following 3 washes in PBS, sections were then incubated with secondary antibodies Alexa Fluor® 647 anti‐Guinea IgG (Invitrogen, A‐21450, 1:800), Alexa Fluor® 594 anti‐Mouse IgG (Invitrogen, A‐11005, 1:800), and Alexa Fluor® 488 anti‐Rabbit IgG (Invitrogen, A‐11034, 1:800) for 2 h at room temperature. Then, after washing in PBS for 3 times, sections were incubated with DAPI (Sigma, D9542, 1:1000) diluted in PBS for 15 min before being sealed with ProLong@Diamond antifade reagent (Invitrogen, P36965) and glass covers. Images were obtained using a fluorescence microscope (Nikon AIR).

### 2.6 Western blotting

Western blot analysis was performed to compare the PD‐1 level in the core lesions and the perilesional tissues. Eight pairs of tissue samples were cut into small pieces, homogenized in lysis buffer (BioVision, CA95035) including protease inhibitors (Roche, 04906837001, 05892970001), and centrifuged at 10,000 × rpm at 4°C for 15 min. The protein concentration of the lysates was determined using a BCA protein assay kit (Thermo Fisher, 23227). The extracts (10 μg) were resolved by 10% SDS‐polyacrylamide gel electrophoresis and electro‐transferred to a polyvinylidene difluoride (PVDF) membrane, which was blocked with 5% skimmed milk in 0.5‰ TBST then incubated for 2 h at room temperature. Then, the PVDF membranes were incubated with primary antibodies rabbit anti‐PD‐1 (Proteintech, 18,106‐1‐AP, 1:500), mouse anti‐GAPDH (Proteintech, 60004‐1‐Ig, 1:3000) in 5% skimmed milk overnight at 4°C, followed by incubation with a horseradish peroxidase‐conjugate second antibodies for 2 h at 37°C. Chemiluminescent signals were generated by using SuperSignal West Femto maximum sensitivity substrate kit (Thermo Fisher, 34095), and the immunoreactive bands were visualized by enhanced chemiluminescence (ProteinSimple).

### 2.7 Quantitative real‐time PCR

Total RNA was extracted from the seven pairs of specimens by using MiniBEST Universal RNA Extraction Kit (Takara, 9767). 1 μg of RNA was reverse transcribed to cDNA using oligo (dT) primer (Takara, RR600B). PCR primers were designed based on NCBI and synthesized by Sangon Biotech (Shanghai, China). The following primers were used: human‐GAPDH, rat‐GAPDH, human‐PDCD1, and rat‐PDCD1. The cycling conditions were as follows: Initial denaturation step was performed at 94°C for 1 min, followed by 40 cycles of denaturation step at 94°C for 30 s, annealing step at 55°C for 30 s, extension step at 72°C for 1 min, and finally the final extension at 72°C for 5 min. Relative quantification was calculated using the 2^−ΔΔCt^ method.

Human‐*PDCD1* (forward: 5′‐CTTCACCTGCAGCTTCTCCA‐3′; reverse: 5′‐CCGCAGGCTCTCTTTGATCT‐3′), rat‐*PDCD1* (forward: 5′‐GATATCCCAGACCCTCACCCA‐3′; reverse: 5′‐CAGCTTCTCTGGCCTCTGACAT‐3′).

### 2.8 Behavioral analysis and video EEG recording in animals

The male C57BL/6 mice and SD rats were randomly divided into saline control group and SHR‐1210 (10 μg) pretreatment group (10 mg SHR‐1210 dissolved in 1000 μL saline). Before intracerebroventricular injection with either saline or SHR‐120, the animals were placed in the plastic cages and acclimatized for at least 30 min. Thirty minutes after intracerebroventricular injection, PTZ (50 mg/kg) was injected intraperitoneally to induce seizures. Epileptic behavior and video EEG were simultaneously recorded for 30–60 min after PTZ administration. For EEG recordings, electrophysiological signals were amplified (×1000) and sampled at 1 kHz (low‐pass filtered at 100 Hz) using NeuroLog System (Digitimer Ltd, Heartford Shire, UK), digitized with CED Micro 1401 (Cambridge Electronic Design, Cambridge, UK), and recorded in a computer using Spike software (version 6.0, Cambridge Electronic Design, Cambridge, UK).

Epileptic behaviors in rodents were scored using the improved five‐graded Racine Score system. Briefly, Racine score 1: facial clonus, head nodding; Racine score 2: tail erection, unilateral hindlimb clonus; Racine score 3: unilateral forelimb clonus, back clonus; Racine score 4: rearing with bilateral forelimb clonus; and Racine score 5: rearing and falling, loss of postural control. The assessment of the seizure severity in an animal was based on the maximal grade observed and the percentage of mice that developed generalized tonic–clonic seizures (GTCs).

### 2.9 Culture of the primary hippocampal cell and transfection

Primary hippocampal neurons were prepared from embryonic day 17–18 Sprague–Dawley rats as we previously reported.^28^ Briefly, the pregnant rat was anesthetized with Urethane (25%, 0.4 mL/kg, i.p.) and the pups were dissected out for tissue preparation. After the dissection of the hippocampus, the tissue was rinsed in cold HBSS and then digested with 0.05% trypsin–ethylenediaminetetraacetic acid for about 20 min at 37°C. Then, DMEM with 10% FBS was used to end the digesting process. Single neurons were collected in the plating medium DF12 (DMEM, 10% FBS, 10% F12) and then planted on the coverslips, which were pre‐coated by poly‐D‐lysine (0.1 mg/mL). After culturing for 1 day, half of the media were changed into neuronal culture media (neurobasal media containing 2 mM GlutaMAX Supplement, 2% B27, and 25 μg/mL penicillin/streptomycin). Ara‐C (2 μM) was added 6–8 days after plating, and cells were fed twice weekly thereafter. All cells were grown at 37°C and in 5% CO_2_. All culture reagents were ordered from Invitrogen. Hippocampal neuronal cultures were transfected with scram‐EGFP or mi*PDCD1*‐EGFP plasmids using the calcium phosphate cell transfection protocol described previously.^28^

### 2.10 Acute slice preparation

Male C57BL/6 mice, aged P28–35, were anesthetized with isoflurane. After decapitation, brains were dissected quickly and placed in ice‐cold NMDG cutting solution containing (in mM) 92 NMDG, 82 NaCl, 2.5 KCl, 1.2 NaH_2_PO_4_, 20 HEPES, 30 NaHCO_3_, 25 Glucose, 5 Na‐ascorbate, 3 Na‐pyruvate, 2 Thicurea, 10 MgSO_4_, 0.5 CaCl_2_, and pH 7.3–7.4 (300–310 mOsM). Slices (250 μm) were collected with a vibratome (Leica Instruments) and maintained in continuously oxygenated artificial cerebrospinal fluid (ACSF) for at least 30 min at 34°C and then at room temperature for 30 min before recording. Individual slices were transferred to a submerged recording chamber visualized with infrared optics microscope (Nikon), where they were perfused continuously with carbogen‐buffered ACSF.

### 2.11 Whole‐cell electrophysiology

In the experiment, standard whole‐cell patch‐clamp recordings were performed using a Multiclamp 700B amplifier (Axon), a Digidata 1440A digitizer (Axon), and pCLAMP 10.3 software (Axon). To record epileptiform burst in primary hippocampal neurons or acquire resting membrane potential (RMP) and input–output (I‐O) curves of hippocampal neurons in CA1, the recording pipettes were pulled from borosilicate glass by pipette puller P‐97 (Sutter Instrument), with the resistance of 3–5 MΩ when filled with intracellular recording solution containing (in mM): 125 K‐gluconate, 10 KCl, 10 Hepes, 10 Tris‐phosphocreatine, 4 Mg‐ATP, 0.5 Na‐GTP. pH was adjusted to 7.2 with 0.5 M KOH and the osmolarity was adjusted at ∼305 mOsm. The bath solution for cultured neurons contained (in mM): 128 NaCl, 30 D‐glucose, 25 HEPES, 5 KCl, 2 CaCl_2_, 1 MgCl_2_. pH was adjusted to 7.3 with 0.5 M NaOH and the osmolarity was adjusted at ∼310 mOsm. The definition of epileptiform bursting was at least five consecutive action potentials overlaying a large depolarization shift, and we considered neurons showing at least two epileptiform bursts within 10 min to be epileptiform neurons. RMP was recorded within the first 10 s after achieving whole‐cell configuration in current‐clamp mode, while the cell is at rest without any holding current. The action potential input–output curve was generated by injecting positive current in current‐clamp mode from −40 to 250 pA at 10‐pA increments for 1000 ms.

To record sodium currents, recording electrodes were filled with an intracellular solution containing (in mM) 110 CsCl, 5 NaCl, 3 MgCl_2_, 1 CaCl_2_, 3 EGTA and 40 HEPES at pH 7.4. The bath solution was consisted of (in mM) 100 NaCl, 40 TEA‐Cl, 3 KCl, 1 MgCl_2_, 1 CaCl_2_, 10 D‐glucose, 10 HEPES, 1 BaCl_2_, 1 CsCl, 2 4‐AP and 0.1 CdCl_2_ bubbled with 95% O_2_–5% CO_2_ at pH 7.2. For steady‐state activation, the peak currents were determined using 50‐ms pulses from −80 mV to 30 mV in 10‐mV steps from a holding potential of −70 mV at 5‐s intervals. Conductance as a function of voltage was obtained from the current–voltage relationship and fitted by the Boltzmann function: G/G_max_ = 1/(1 + exp [V − V1/2)/k] to determine the voltage midpoint (V1/2). For steady‐state inactivation, cells were held at −140 mV, and the test potential was from −140 to 20 mV for 500 ms at 10‐mV increments. A second pulse to −10 mV for 50 ms was used to assess channel availability. The normalized current was plotted against voltage, and steady‐state inactivation curves were also fitted with the Boltzmann equation I/I_max_ = 1/(1 + exp [V − V1/2)/k] to determine the voltage midpoint (V1/2).

### 2.12 Co‐immunoprecipitation

Human brain tissues were lysed in the NP‐40 lysis buffer containing PMSF (1 mM), inhibitor cocktail, and phosphatase inhibitor. Lysates were centrifuged at 15,000 g for 10 min, and the supernatant was transferred into new tubes. A total of 10 μg PD‐1 or Nav1.6 antibodies were added to Protein G Dynabeads for cross‐linking for 1 h, then mixed with the supernatant above, and incubated overnight at 4°C to form an antigen–antibody compound on a revolving rotor. After 3 washes using washing buffer, the compound was eluted and separated. The supernatant was mixed with 5× loading buffer and heated for 5 min at 95°C for PD‐1 WB or 2× loading buffer without heating for Nav1.6 WB.

### 2.13 Statistical analyses

Most data in figures were presented as mean ± S.E.M., as indicated in the Figure legends. For the figures regarding the maximum Racine score in two groups, the median and interquartile range (IQR) was displayed. Statistical analysis of results was completed with Prism GraphPad 8.0. Biochemical, behavioral, and electrophysiology data were analyzed using paired Student's *t*‐test, unpaired Student's *t*‐test, one‐way ANOVA (multiple groups), Mann–Whitney test (two groups of ordinal variables), or Fisher's exact test (two groups of two categorical variables). *p* < 0.05 was considered to indicate a statistically significant difference. Data are presented in the mean ± SEM. *p < 0.05, **p < 0.01, ***p < 0.001.

## 3 RESULTS

### 3.1 PD‐1 expression is elevated in the lesion tissue of patients with epilepsy

**FIGURE 1:**
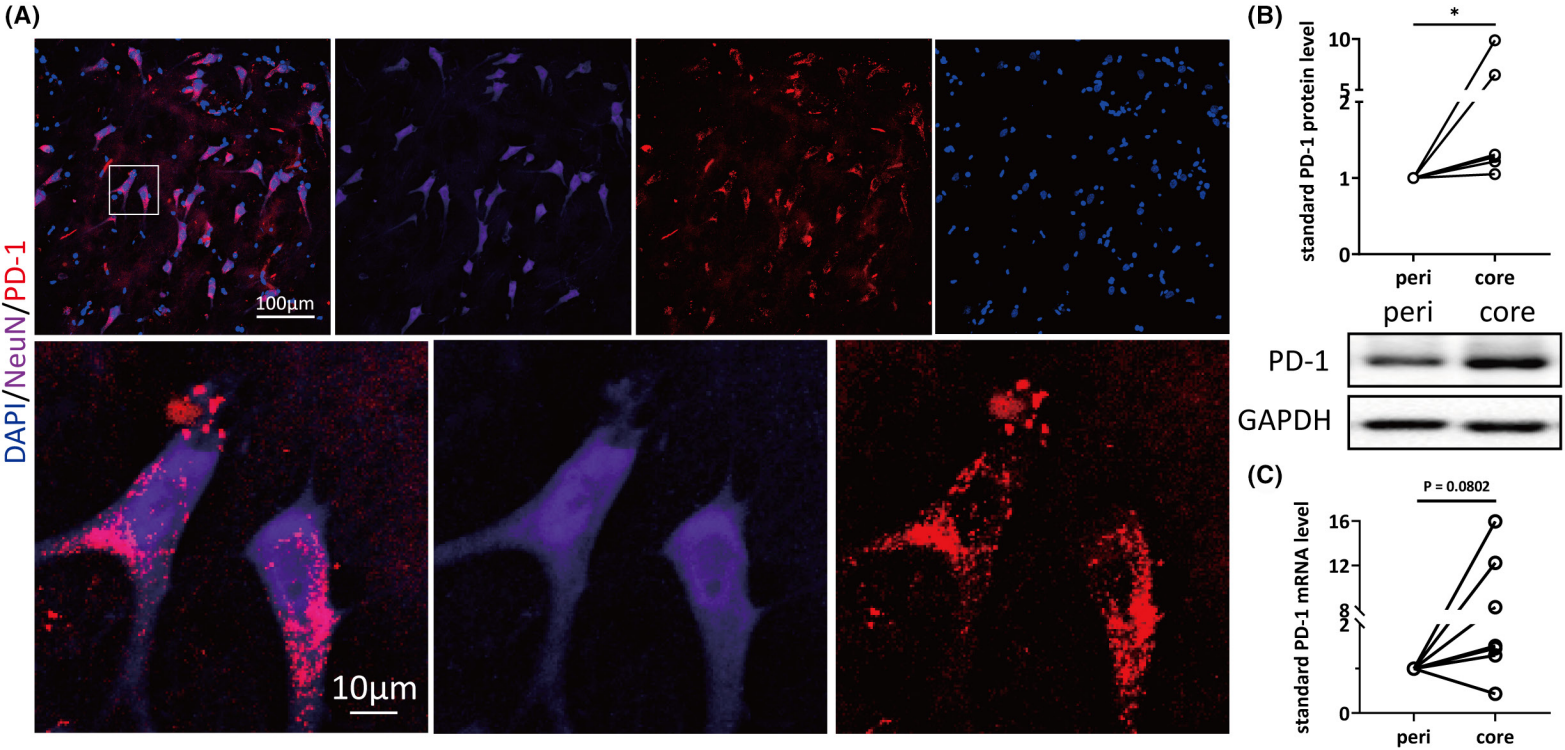
The expression level of programmed cell death protein 1 (PD1) is elevated in the lesion tissue of epilepsy patients. (A) Immunostaining for PD‐1 and NeuN in focal tissue from patient #5 and higher magnification of the cell shown in the white square in the upper row. (B) Quantification of PD‐1 protein levels in focal and perifocal tissues from eight patients with refractory epilepsy and representative immunoblot bands of PD‐1. (C) Quantification of PD‐1 mRNA levels in focal and perifocal tissues from seven patients with refractory epilepsy. Statistical analysis: paired Student's *t*‐test. *p < 0.05.

To explore the association between PD‐1 and epilepsy, we first confirmed that the PD‐1 protein is expressed in human neurons by performing immunohistochemistry. We collected focal tissues responsible for seizure onset as foci (lesion cores) and surrounding surgically resected tissues (perifocal tissues) as controls from patients who underwent lesion resection due to drug‐resistant epilepsy. Detailed information about these samples is listed in Table S1. Double staining results revealed that PD‐1 was colocalized with a neuronal marker (Figure 1A) but was barely colocalized with astrocytic and microglial markers (Figure S1). Given the fact that there was neuronal PD‐1 expression in human brain tissues, we then analyzed the protein expression and mRNA level of PD‐1 in surgically resected specimens. Eight pairs of cortical tissues from patients with refractory epilepsy were used for WB, and seven pairs were used for RT–qPCR. As shown in Figure 1B, the protein expression of PD‐1 was significantly increased (*n* = 8, *p* = 0.0288) in the lesion core compared with perifocal tissues. Consistent with the results obtained by WB, real‐time qPCR showed a tendency for PD‐1 mRNA levels to be increased in the lesion core compared with the perifocal control tissues (*n* = 7, *p* = 0.0802) (Figure 1C). The data presented here revealed upregulated expression of PD‐1 in the pathological lesion core compared with perifocal tissues at both the mRNA and protein levels, suggesting that elevated expression of PD‐1 might be implicated in the pathology of epilepsy.

### 3.2 Anti‐PD‐1 treatment has anti‐epileptic effects both in vivo and in vitro

**FIGURE 2:**
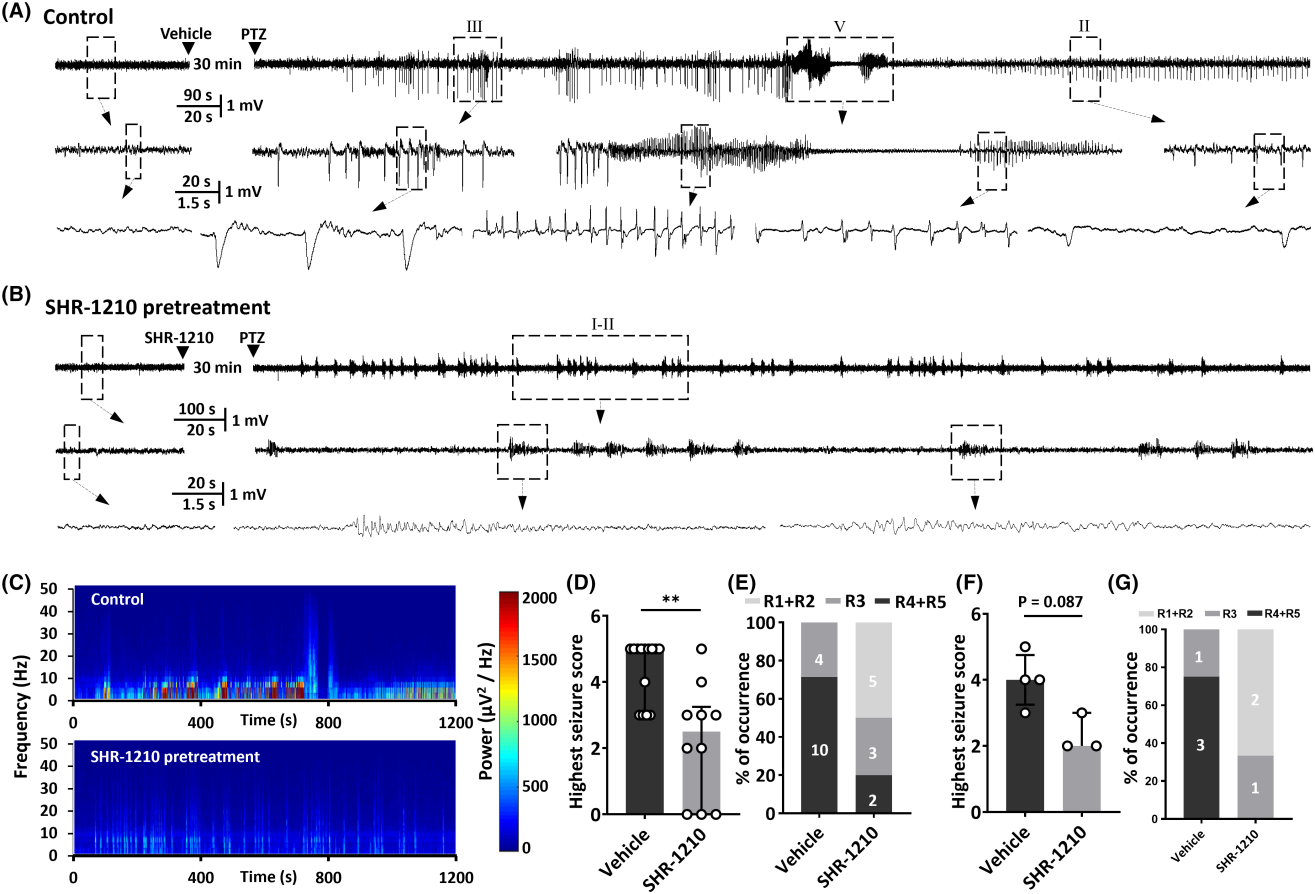
Anti‐PD‐1 treatment has an anti‐epileptic effect in rodent models. (A, B) Representative traces of EEG recordings from pentylenetetrazol (PTZ)‐induced epilepsy model rats with different Racine scores (upper) pretreated with either vehicle (A) or the anti‐PD‐1 antibody SHR‐1210 (B). (C) Corresponding wavelet spectrograms of the vehicle control and SHR‐1210 pretreatment groups. (D–G) Histograms showing the seizure score following PTZ administration with or without SHR‐1210 pretreatment. The highest seizure score of mice (D) and rats (F) after PTZ treatment (Figures displaying the median and IQR). The percentage of mice (E) and (G) rats with PTZ‐induced seizures showing a Racine score of 1–5. Statistical analysis: Mann–Whitney *U*‐test. (D, F). **p < 0.01.

Based on the finding that neuronal PD‐1 was abnormally upregulated in epileptic tissues, we wondered about the function of neuronal PD‐1. To learn whether the function of PD‐1 can affect seizure severity, we examined the effect of blocking PD‐1 activity in vivo on electroencephalograms and the behavior of rodents with acute seizures induced by PTZ, a classical chemoconvulsant.^29^, ^30^ We quantified the percentage of animals that developed GTCs and the average highest seizure score to determine the severity of seizure. PD‐1 was acutely blocked by intracerebroventricular injection of the PD‐1 monoclonal antibody SHR‐1210 (10 μg/1 μL). The raw EEG traces shown in Figure 2A,B and the results of power spectral analysis shown in Figure 2C are representative data from one pair of rats treated with or without the anti‐PD‐1 antibody. The histograms of the percentage of animals that reached the highest seizure stage and the average highest seizure score in either species, which are shown in Figure 2D–G indicate that anti‐PD‐1 treatment successfully prevented PTZ‐induced seizures both in mice (vehicle: 4.6 ± 0.3, *n* = 10; SHR‐1210: 2.2 ± 0.6, *n* = 14; *p* = 0.0014) and in rats (vehicle: 4.0 ± 0.4, *n* = 4; SHR‐1210: 2.3 ± 0.3, *n* = 3; *p* = 0.0857).

**FIGURE 3:**
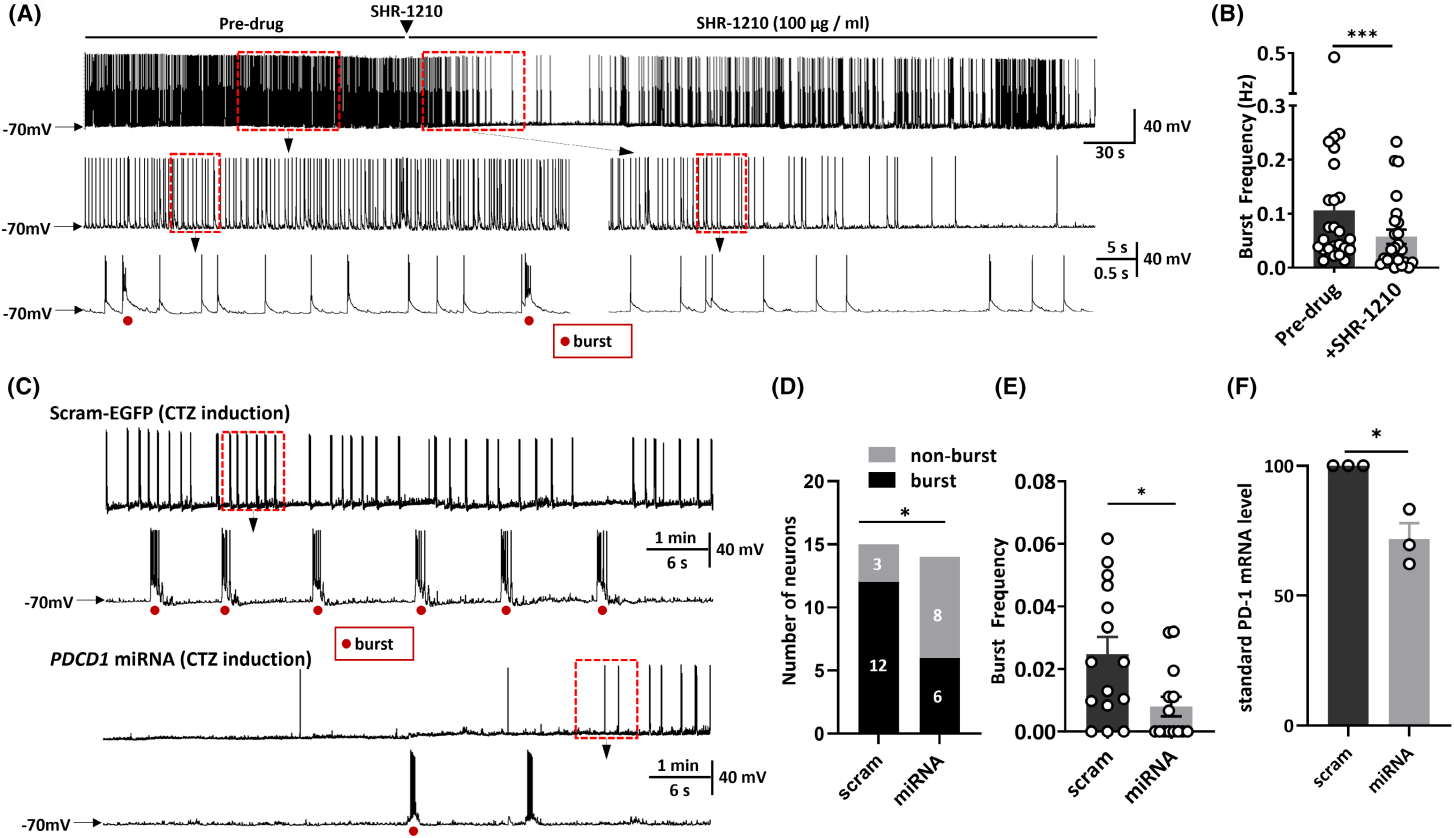
Both pharmacological blockade and genetic knockdown of programmed cell death protein 1 (PD‐1) can alleviate epileptiform bursting in epileptic hippocampal neurons. (A) Representative neuronal activities and enlarged traces before and after perfusion of ACSF containing SHR‐1210. (B) The burst frequency of hippocampal neurons recorded before and after perfusion of ACSF containing SHR‐1210. (C) Representative neuronal activities and enlarged traces in the scrambled and mi‐*PDCD1* groups. (D) The proportion of neurons showing epileptiform burst activity in the scrambled and mi‐*PDCD1* groups. (E) The burst frequency of hippocampal neurons in the scrambled and mi‐*PDCD1* groups. (F) Quantification of PD‐1 mRNA levels in the scrambled and mi‐*PDCD1* groups. The red dots indicate bursting activity induced by cyclothiazide (CTZ). Statistical analysis: paired Student's *t*‐test. (B), unpaired Student's *t*‐test. (E, F), and Fisher's exact test (D). The data are the means ± SEMs. *p < 0.05, ***p < 0.001.

Given the finding that PD‐1 blockade could protect animals from seizures, combined with the fact that PD‐1 can regulate the function of ion channels,^14^, ^15^, ^17^ we hypothesized that PD‐1 signaling can alter the electrical activity of neurons, thus playing a potential role in the generation of seizures. Therefore, to assess whether the excitability of neurons is altered by PD‐1 blockade, we performed patch‐clamp recordings on cultured primary hippocampal neurons. The electrical activities of hippocampal neurons were assessed via whole‐cell patch recording after coincubation with CTZ (5 μM, 48 h), a convulsant drug that induces robust epileptiform activity in neurons.^31^ This in vitro epileptic model was established and characterized in our previous studies.^31^, ^32^, ^33^ The definition of an epileptiform burst was at least five consecutive action potentials overlaying a large (>10 mV) depolarization shift lasting at least 300 ms,^31^ and we considered neurons showing at least two of these epileptiform bursts within a 10 min recording period to be neurons showing epileptiform activity. Coincubation of cultured primary hippocampal neurons with CTZ led to the transformation of neurons to epileptiform bursting neurons with robust synchronized epileptiform burst discharges, consistent with as previous reports,^31^, ^32^, ^33^ with an average frequency of 0.106 ± 0.022 Hz (*n* = 25, Figure 3A,B). To test whether anti‐PD‐1 treatment is capable of altering epileptiform activity in this in vitro model, we applied SHR‐1210 (100 μg/mL), an anti‐PD‐1 antibody, by acute perfusion. After perfusion with ACSF containing SHR‐1210, the frequency of neuronal epileptiform burst discharges from the same neuron was substantially reduced to 0.057 ± 0.013 Hz (*n* = 25, *p* = 0.0002) (Figure 3A,B). In addition, we compared the burst frequency before and after continuous perfusion of ACSF containing no drug and found that this treatment did not have the same rundown effect over the same period as drug application (Figure S2). Moreover, since the SHR‐1210 used in the current study was a monoclonal antibody produced and gifted by Hengrui Pharmaceutical (China) for experimental use before it was marketed for clinical use, we also tested the effect of an FDA‐approved human anti‐PD‐1 monoclonal antibody (nivolumab). As shown in Figure S3, the burst frequency after perfusion with nivolumab (100 μg/mL) was 0.013 ± 0.006 Hz, which represented a substantial decreased from that observed before nivolumab perfusion (0.071 ± 0.025 Hz) (*n* = 6, *p* = 0.0362).

To further demonstrate that PD‐1 functional deficits can attenuate CTZ‐induced neuronal bursting, we knocked down the expression of PD‐1 mRNA in cultured primary hippocampal neurons by transfecting them with *PDCD1*‐miRNA labeled with EGFP (mi*PDCD1*‐EGFP) or scram‐EGFP, serving as a control group where scrambled microRNA (miRNA) tagged with an enhanced green fluorescent protein (EGFP) marker was used to eliminate interference from transfection and the operation. We first verified the efficiency of PD‐1 knockdown by applying RT–qPCR and confirmed that the mRNA level was significantly reduced (scram: 100.0 ± 0.0%, *n* = 3 v.s. mi*PDCD1*: 71.7 ± 6.2%, *n* = 3; *p* = 0.01) (Figure 3F). We found that after CTZ incubation, both the proportion of neurons showing epileptiform burst activity and the average epileptiform burst frequency were significantly decreased in neurons transfected with mi*PDCD1* compared with neurons transfected with the control scrambled‐EGFP plasmid. Furthermore, 6/14 neurons in the mi*PDCD1* group and 12/15 in the scrambled group showed epileptiform burst activity (*p* = 0.03) (Figure 3D,E), and the burst frequency of all the neurons tested was 0.008 ± 0.003 Hz (*n* = 15) in the mi*PDCD1* group and 0.025 ± 0.005 Hz (*n* = 14) in the scram‐EGFP group (*p* = 0.015) (Figure 3F). Considering these results combined with those reported above, we concluded that PD‐1 in primary hippocampal neurons is functional and that both acute blockade of PD‐1 by a monoclonal antibody and genetic knockdown of PD‐1 can markedly alleviate neuronal epileptiform activity and decrease the burst frequency of action potentials directly.

### 3.3 PD‐1 regulates sodium channel function in neurons showing epileptiform activity

**FIGURE 4:**
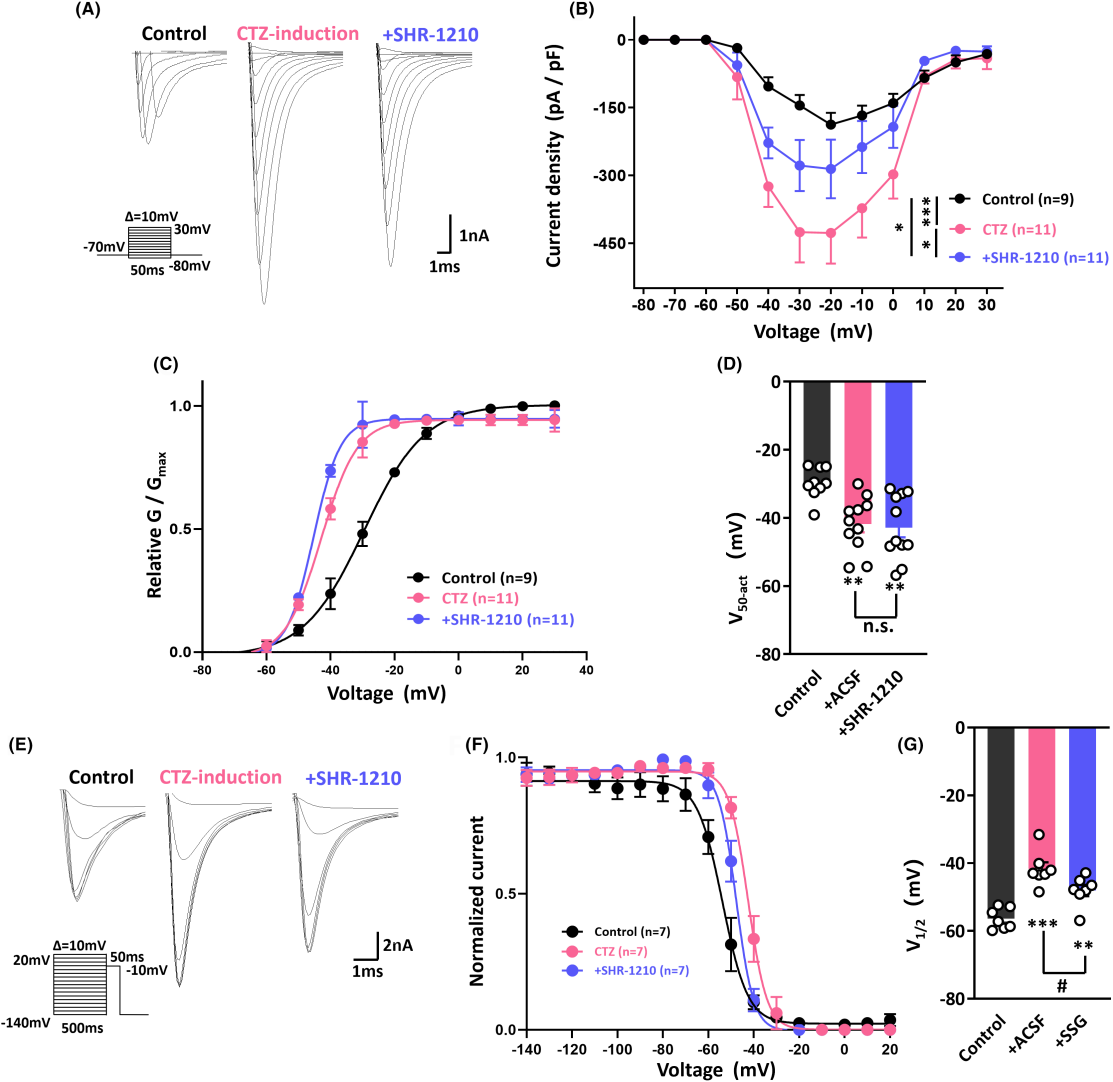
Programmed cell death protein 1 (PD‐1) regulates sodium channel function in neurons showing epileptiform activity. (A) Diagram of the voltage‐clamp protocol involving depolarization of the membrane potential from −80 to 30 mV in 10 mV steps and typical types of sodium currents observed during application of the voltage‐clamp protocol when determining the steady‐state activation kinetics of sodium channels. (B) The current density versus voltage relationships obtained was by averaging the data from the control, cyclothiazide (CTZ)‐treated, and CTZ+ SHR‐1210 perfusion groups when determining the steady‐state activation kinetics of sodium channels. (C) Plots of the relative conductance (G/G_max_) versus membrane potential during sodium channel steady‐state activation in the control, CTZ induction, and CTZ + SHR‐1210 perfusion groups upon fitting with the Boltzmann equation. (D) Statistical chart showing the potential at which half of the sodium channels opened, which was determined when calculating the steady‐state activation kinetics of sodium channels in the control, CTZ induction, and CTZ + SHR‐1210 perfusion groups. (E) Diagram of the voltage‐clamp protocol involving depolarization from −140 to 20 mV in 10‐mV steps and typical test‐pulse currents for potentials between −140 and +20 mV observed during application of the voltage‐clamp protocol when determining the steady‐state inactivation kinetics of the sodium channel. (F) Plots of the normalized current (I/I_max_) versus membrane potential of inactivated sodium channels after fitting with the Boltzmann equation. (G) Histogram of the potential at which 50% of the sodium channels were closed, which was determining when assessing the steady‐state inactivation kinetics of sodium channels in the control, CTZ induction, and CTZ + SHR‐1210 groups. Statistical analysis: ordinary one‐way ANOVA (D, G). The data are the means ± SEMs. *p < 0.05, **p < 0.01, ***p < 0.001.

Considering the protective effect of anti‐PD‐1 treatment on neuronal epileptiform bursts, we wondered what the underlying mechanism is. It is well accepted that sodium channels play an important role in initiating action potentials in neurons and their dysfunction can cause inherited epilepsy.^34^ Additionally, it has been reported that PD‐1 is a potential regulator of sodium currents.^15^ Therefore, to further investigate the mechanism by which SHR‐1210 diminishes convulsant‐induced overexcitation of hippocampal neurons, we examined the effects of PD‐1 inhibition on the kinetics of voltage‐activated sodium channels in hippocampal neurons. The steady‐state activation kinetics of sodium channels were first tested under control conditions, upon treatment with CTZ and following SHR‐1210 perfusion. As shown in Figure 4A, sodium currents were determined by applying the voltage‐clamp technique; representative types of sodium currents observed during application of these three treatment protocols are illustrated. Analysis of current density versus voltage relationships from the averaged data showed that the peak current density in the CTZ coincubation group was significantly higher than that in the control group and that this change could be reversed by SHR‐1210 perfusion (Figure 4B). Figure 4C presents plots of the relative conductance (G/G_max_) versus membrane potential in these three groups. These data were fitted with the Boltzmann equation, and the results revealed that in neurons with epileptiform discharges, the membrane potential required to elicit opening of half of the sodium channels was significantly shifted from −29.7 ± 1.5 mV (*n* = 9) to −41.8 ± 2.4 mV (*n* = 11, *p* = 0.0047), suggesting that the CTZ‐induced neurons are more easily excited than control neurons. The half‐activation potential of CTZ + SHR‐1210‐treated neurons was −42.9 ± 2.8 mV (*n* = 11), which was not different than that of CTZ alone‐treated neurons, indicating that treatment with the anti‐PD‐1 antibody SHR‐1210 did not affect the opening status of overactivated sodium channels in neurons induced by CTZ (Figure 4D).

Thus, we further tested the steady‐state inactivation kinetics of sodium channels; typical test‐pulse currents for potentials between −140 and +20 mV are shown in Figure 4E. The data were fitted with the Boltzmann equation, and as illustrated in Figure 4F, we revealed a depolarizing shift of 15 mV for sodium channels in the CTZ‐treated group compared with the control group; that is, the midpoint potential for the normalized current–voltage curve was −41.7 ± 1.9 mV in the CTZ group (*n* = 7) compared with −56.4 ± 1.2 mV (*n* = 7, *p* < 0.001) in the control group. It is quite plausible that the convulsant effect of CTZ on neurons might have partially been due to the loss of sodium channel blockage. However, the midpoint potential after the application of SHR‐1210 was decreased to −48.1 ± 1.7 mV (*n* = 7, *p* = 0.0329), which significantly differed from the midpoint potential of the CTZ alone group, suggesting that blockade of PD‐1 by SHR‐1210 could reverse the epileptogenic effect of CTZ via sodium channel blockage (Figure 4G).

### 3.4 PD‐1 and SHP‐1 regulate neuronal excitability together

**FIGURE 5:**
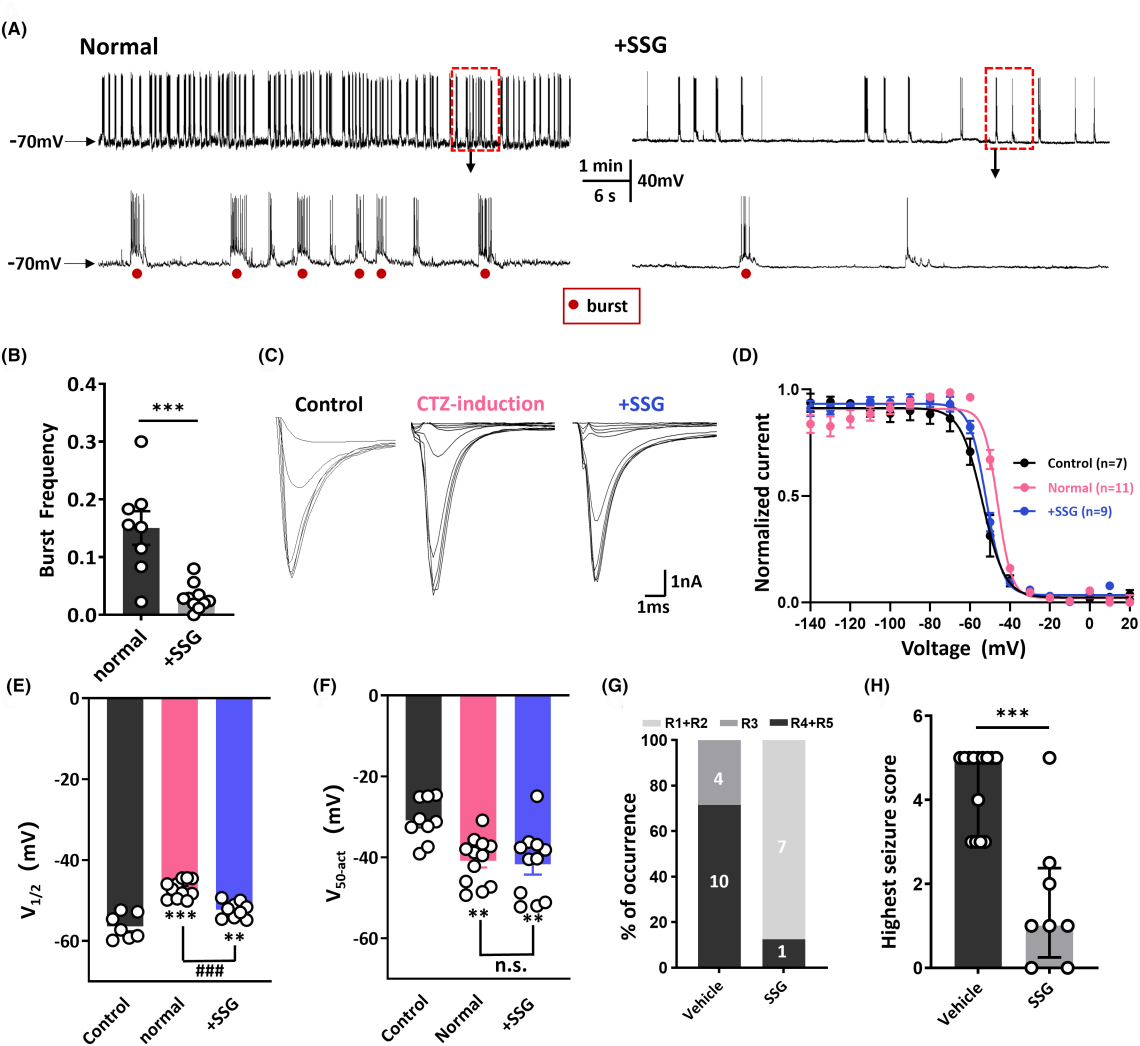
Programmed cell death protein 1 (PD‐1) and SHP‐1 regulate neuronal excitability together. (A) Representative neuronal activities and enlarged traces recorded in normal intracellular solution and the intracellular solution containing SSG. (B) The burst frequency of hippocampal neurons recorded in the two groups in (A). (C) Diagram of the voltage‐clamp protocol involving depolarization from −140 to 20 mV in 10‐mV steps and typical test‐pulse currents for potentials between −140 and +20 mV observed during application of the voltage‐clamp protocol when determining the steady‐state inactivation kinetics of sodium channels in the control, cyclothiazide (CTZ)‐incubated, and SSG‐treated groups. (D) Plots of the normalized current (I/I_max_) versus membrane potential of inactivated sodium channels in the three groups in (D) after fitting with the Boltzmann equation. (E) Histogram of the potential at which 50% of the sodium channels were closed when determining the steady‐state inactivation kinetics of sodium channels in the three groups. (F) Statistical chart of the potential at which 50% of the sodium channels were open in the control, CTZ‐incubated, and SSG‐treated groups when determining the steady‐state activation kinetics of sodium channels. (G) The percentage of PTZ‐treated animals showing Racine scores 1–5 in the control group and SSG pretreatment group. (H) The highest seizure score (the median and IQR) in the control group and SSG pretreatment group. The red dots indicate bursting activity induced by CTZ. Statistical analysis: unpaired Student's *t*‐test (B), Mann–Whitney *U*‐test (H), and ordinary one‐way ANOVA (E, F). **p < 0.01, ***p < 0.001.

The ability of PD‐1 to perform its functions is dependent on the recruitment of SHP‐1.^17^, ^35^ Therefore, we hypothesized that PD‐1 acts together with SHP‐1 to regulate neuronal excitability. To address our hypothesis, we applied SSG, a validated inhibitor of SHP‐1,^15^, ^36^, ^37^ to the intracellular solution when performing patch‐clamp recordings on primary cultured hippocampal neurons. As expected, the CTZ‐induced epileptiform burst frequency (0.151 ± 0.029 Hz, *n* = 8) was significantly decreased to 0.03 ± 0.007 Hz (*n* = 10; *p* < 0.001) by acute application of SSG (Figure 5A,B). We then examined the effects of SSG on the kinetics of voltage‐activated sodium channels as described above. We determined the midpoint potential of steady‐state inactivation and various test‐pulse currents for potentials between −140 and +20 mV are shown in Figure 5C. After fitting the data with the Boltzmann equation (Figure 5D), it was clear that the midpoint potential of inactivation sodium currents in CTZ‐induced neurons was significantly depolarized by approximately 10 mV (control: −56.4 ± 1.2 mV, *n* = 7 vs. CTZ: −47.1 ± 0.7 mV, *n* = 11; *p* < 0.001), and intracellular SSG application significantly reversed this depolarization (−52.3 ± 0.7 mV, *n* = 9; *p* < 0.001) (Figure 5E). In contrast, fitting the steady‐state activation data with the Boltzmann equation revealed hyperpolarization of the half‐activation potential by 10 mV in CTZ‐induced neurons (40.8 ± 1.7 mV, *n* = 12) compared to the control group (−30.9 ± 1.8 mV, *n* = 9; *p* = 0.0061); however, similar to anti‐PD‐1 antibody treatment, intracellular SSG application did not alter this change in epilepsy‐related sodium channel steady‐state activation (41.7 ± 2.6 mV, *n* = 11) (Figure 5F).

Furthermore, we found that acute blockade of SHP‐1 by intracerebroventricular injection of SSG successfully decreased the incidence of GTCs and the average highest seizure score following PTZ (50 mg/kg, i.p.) injection in mice (Figure 5G,H). The average highest seizure score evoked by PTZ was 4.6 ± 0.3 (*n* = 14) in the control group compared to 1.6 ± 0.6 (*n* = 8) in the SSG group (*p* < 0.001) (Figure 5H). The similar effects of pharmacological blockade of PD‐1 and that of SHP‐1 on seizure severity with suggested that PD‐1 and SHP‐1 likely act together to regulate seizure activity.

### 3.5 PD‐1 is coexpressed with SHP‐1 and Nav1.6

**FIGURE 6:**
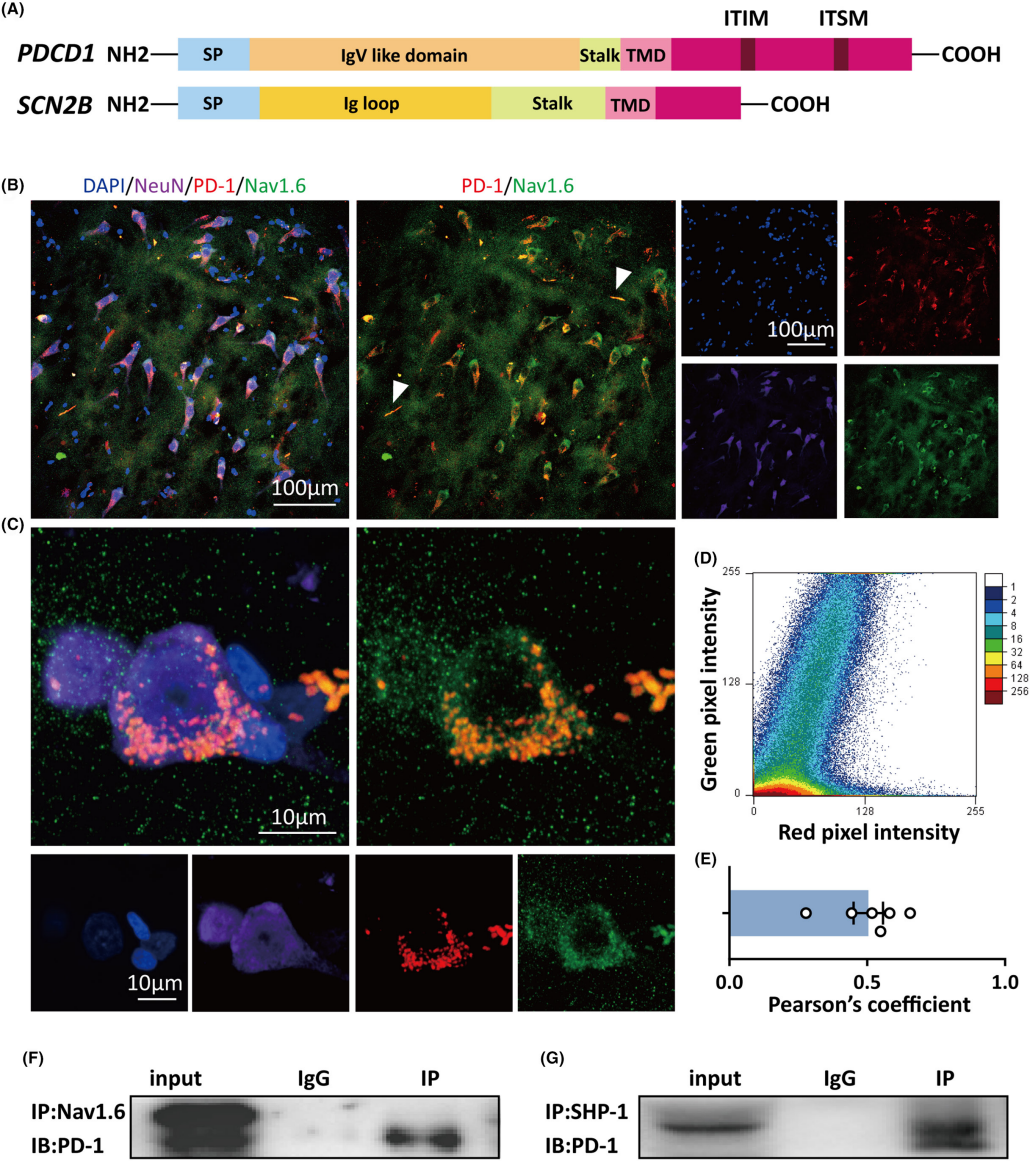
Programmed cell death protein 1 (PD‐1) is coexpressed with Nav1.6. (A) The protein structures of PD‐1 and sodium channel subunit beta‐2. (B) Immunostaining for PD‐1 and Nav1.6 in brain tissue from patient #5. The white arrows point to the axon initial segment. (C) High‐magnification images of immunostaining for PD‐1 and Nav1.6 in brain tissue from patient #8. (D) Scatterplots showing fluorescence colocalization of PD‐1 with Nav1.6 in B. (E) Measurement of the Pearson's correlation coefficient of PD‐1 and Nav1.6. (F) Representative immunoblot bands of PD‐1 coprecipitated by an anti‐Nav1.6 antibody in human brain lysates. (G) Representative immunoblot bands of PD‐1 coprecipitated by an anti‐SHP‐1 antibody in human brain lysates. ITIM, immunoreceptor tyrosine‐based inhibitory motif; ITSM, immunoreceptor tyrosine‐based switch motif; SP, signal peptide; TMD, transmembrane domain.

Given that blockade of either PD‐1 or SHP‐1 impacted the kinetics of sodium channels, it is conceivable that there is an association between the PD‐1 complex and sodium channels. Great strides have been made in understanding the structure of PD‐1, which is a type I transmembrane glycoprotein consisting of a single immunoglobulin variable region (IgV)‐like domain in the N‐terminus, a transmembrane domain, and a cytoplasmic tail containing tyrosine‐based signaling motifs.^38^ Due to its structure and its regulatory effect on sodium channel kinetics, PD‐1 reminds us of VGSC β subunits. VGSC β subunits are also type I transmembrane glycoproteins featuring an extracellular N‐terminal domain with homology to V‐type immunoglobulin (Ig) loops, a transmembrane segment, and a C‐terminal tail (Figure 6A).^39^ All these domains of VGSC β subunits are capable of interacting with VGSC α subunits and modulating α subunit gating and cell surface expression.^40^ Inspired by the idea that PD‐1 might act as a potential auxiliary subunit of VGSCs, we next investigated whether there is a structural interaction between PD‐1 and VGSC α subunits.

There are nine genes encoding α‐subunits, among which Nav1.1, Nav1.2, and Na1.6 are prevalent in the CNS.^41^ Nav1.6 is the dominant sodium channel in the adult brain and is widely distributed on the axon initial segment (AIS) of principal excitatory neurons and inhibitory interneurons, which is responsible for the initiation and propagation of action potentials.^42^ In addition, Nav1.6 has been found to be strongly associated with the onset of seizure activities.^43^, ^44^, ^45^ Therefore, we utilized Nav1.6 as our major target and examined the interaction between PD‐1 and Nav1.6 by performing immunohistochemistry and co‐IP. We coimmunostained for Nav1.6 and PD‐1 in tissues from patients with refractory epilepsy. Immunofluorescence and confocal imaging revealed PD‐1 puncta and a high degree of colocalization between Nav1.6 and PD‐1 on human neurons (Figure 6B,C). The results of colocalization analysis of PD‐1 and Nav1.6 by immunofluorescence are shown in the scatterplot in Figure 6D, and Pearson's correlation coefficient (PPC) was 0.5 ± 0.05 (*n* = 6), suggesting fairly strong colocalization of PD‐1 with Nav1.6 (Figure 6E). Interestingly, colocalization of Nav1.6 with PD‐1 in the AIS could be seen and is indicated by the white arrows in Figure 6B. Moreover, a co‐IP experiment also demonstrated that PD‐1 was coprecipitated by an anti‐Nav1.6 antibody in human brain lysates (Figure 6F). Moreover, we performed co‐IP to validate that there was an interaction between PD‐1 and SHP‐1 (Figure 5G). The results indicated an extremely high possibility that there is a close interaction between the PD‐1 complex and Nav1.6 in human brain tissue from patients with epilepsy. Therefore, it is possible that PD‐1 impacts the inactivation kinetics of sodium channels via its C‐terminal tail, thus playing a vital role in regulating neuronal excitability.

## 4 DISCUSSION

PD‐1 is a typical immune checkpoint modulator that is recognized as an emerging target for cancer immunotherapy. However, the role of PD‐1 in the CNS has begun to attract the attention of researchers. PD‐1 has been shown to play a crucial role in neurological disorders such as pain, glioblastoma, Alzheimer's disease, and ischemic stroke.^9^, ^10^, ^14^, ^46^ Recent studies have also elucidated the unique function of neuronal PD‐1, which is different from that of PD‐1 in immune cells.^15^, ^16^ To our knowledge, however, no studies have identified a relationship between neuronal PD‐1 and epilepsy. Notably, PD‐1 can modulate the function of ion channels and affect neuronal activity.^15^, ^17^ Therefore, there is a pressing need to investigate the role of PD‐1 in epilepsy. As a first step to address the relationship between PD‐1 and epilepsy, we first analyzed the expression of PD‐1 in surgically resected specimens from patients with intractable epilepsy. As evidenced by WB, the PD‐1 protein level was significantly upregulated in focal tissues responsible for seizure onset compared to the perifocal area in the same patient. However, although there is a tendency of increased mRNA levels, the lack of statistical significance is likely due to the considerable variation among the samples. There may be two main reasons for the large variation in mRNA levels. First, the inherent differences of the tissues resulting from severity and duration of epilepsy, age, and underlying health conditions of the patients. Second, due to the extended storage duration of our samples, mRNA degradation may have occurred to varying extents. The data presented here are the first to our knowledge to demonstrate that neuronal PD‐1 was upregulated in human tissues with epilepsy‐related pathological changes. Actually, recent research has shown that the expression of PD‐1 on Treg lymphocytes was much higher in patients with intractable epilepsy than the control, which indicates that Treg cells may experience upregulation of PD‐1 expression in response to recurrent epileptic seizures.^47^ Similarly, our findings indicated that neurons could manifest a similar upregulation in PD‐1 expression, though the underlying mechanisms have yet to be elucidated.

Next, in order to figure out whether the neuronal PD‐1 might affect the onset of seizures and to investigate the potential effect of PD‐1 monoclonal antibody on seizure, we employed PTZ‐induced seizure models. The results showed that the intracerebroventricular administration of the anti‐PD‐1 monoclonal antibody SHR‐1210, now referred to as camrelizumab, was efficacious in ameliorating severe seizures in rodents subjected to PTZ. Moreover, by applying CTZ‐induced neuronal epilepsy model, we also observed that the application of SHR‐1210 effectively reduced the frequency of epileptiform bursting. That is, anti‐PD‐1 treatment was shown to have a protective effect against seizure both in vivo and in vitro.

The mechanism underlying the anti‐epileptic effect of SHR‐1210 involves alteration of the kinetics of voltage‐activated sodium channels. VGSCs play a crucial role in the regulation of neuronal excitability. Activation and inactivation, the two fundamental properties of sodium channels, are necessary for sodium channels to carry out their physiological roles.^48^, ^49^ In addition, the midpoint potential of the inactivation‐voltage relationship can reflect the resting membrane potential of a neuron to a certain extent.^50^ Therefore, the electrophysiological properties of inactivated sodium channels can significantly influence the electrical stability of neurons. Sodium channel mutations were reported to be associated with human epilepsy, causing subtle changes in inactivation characteristics in vitro.^51^ Here, we show that blockade of PD‐1 can hyperpolarize the midpoint potential of sodium channels following depolarization by CTZ, preventing the epileptogenic effect of CTZ via sodium channel blockage. The relationship between PD‐1 and SHP‐1 led us to explore the function of SHP‐1 in the regulation of neuronal excitability. The capability of PD‐1 to block T‐cell activation is dependent on the recruitment of SHP‐1, and the regulatory effect of PD‐1 on neuronal excitability in DRG neurons is also associated with SHP‐1.^15^, ^17^, ^35^ Therefore, we tested the effect of SHP‐1 blockade on epileptiform bursting and sodium channel kinetics in CTZ‐treated hippocampal neurons as well as PTZ‐treated mice, and we revealed that blockade of SHP‐1 by SGG has the same effect as SHR‐1210.

We then asked what the mechanism underlying the effect of PD‐1 and SHP‐1 blockade on sodium channels is? The structure of PD‐1 reminded us of that of VGSC β subunits, which play a crucial role in the regulation of alpha subunit expression and function. Moreover, according to the crystal structure of the Nav1.4‐β1 complex, the C‐terminal tail of Nav1.4 may modify inactivation through an allosteric blocking mechanism.^39^ Therefore, we proposed that both blockade of PD‐1 by monoclonal antibodies and irreversible inhibition of SHP‐1 by SSG affect the topology of the PD‐1 complex, thus modifying sodium channel inactivation. We next investigated whether PD‐1 interacts structurally with VGSC α subunits like it does with auxiliary β subunits. Among the VGSC α subunits in the CNS, Nav1.6, which is prevalent in the adult brain and widely distributed on the AIS and soma of principal excitatory neurons, contributes to the initiation and propagation of action potentials.^23^ Mutations in Nav1.6 have been reported to be associated with epileptic encephalopathy characterized by developmental delay.^26^, ^52^ Therefore, we next performed coimmunostaining for Nav1.6 and PD‐1 in human brain tissues from patients with drug‐resistant epilepsy. Immunofluorescence suggested that Nav1.6 colocalized with PD‐1. Existing studies have shown that the function of a protein is often modulated by other proteins that interact with it. Therefore, we assumed that there is an interaction between PD‐1 and Nav.16 and that this interaction underlies the regulatory effect of PD‐1 on sodium channels. We tried to validate this protein–protein interaction by performing co‐IP, a classical technique for identifying protein–protein interactions that has been used in numerous experiments.^53^ Further evidence from co‐IP indicated an interaction among PD‐1, SHP‐1, and Nav1.6, which provides a convincing explanation for the role of PD‐1 in sodium channel blockade and neuronal excitability regulation.

PD‐1 was assumed to play an essential role in neuromodulation via PD‐1/SHP‐1 signaling and downstream modulation of ion channels in recent research.^54^ However, our conclusion seems to be contrary to that of other researchers, as previous studies have indicated that activation of the PD‐1 pathway suppresses the function of sodium channels in DRG neurons, thus inhibiting neuronal excitability. For example, PD‐1 activation in nociceptive DRG neurons suppresses sodium currents by phosphorylation of SHP‐1.^15^ Moreover, recent research revealed that the presence of functional PD‐1 on hippocampal CA1 neurons had an inhibitory effect on neuronal excitability, as both knockout and blockade of PD‐1 made neurons more excitable, contributing to improvements in memory and learning in experimental animals.^55^ It is possible that the effects of PD‐1 blockade on DRG neurons are different from the effects of PD‐1 blockade observed in our study. First, different types of sodium channels are distributed in different types of neurons. DRG neurons mainly express Nav1.7, Nav1.8, and Nav1.9, whereas hippocampal neurons mainly express Nav1.2 and Nav1.6.^56^, ^57^ Therefore, it is conceivable that PD‐1 may play distinct roles in regulating neurons expressing different types of sodium channels. Second, we do not consider PD‐1 as a signal‐transducing receptor, but it regulates the electrophysiological properties of sodium channels through direct interaction with sodium channels. Here, we propose that PD‐1, Nav1.6, and SHP‐1 form a complex and that blockade of both PD‐1 and SHP‐1 can affect sodium channel inactivation. To date, researchers have found that anti‐PD‐1 treatment could elicit ERK phosphorylation via SHP‐1 signaling and abolish currents mediated by GABA in cortical neurons.^14^ However, whether PD‐1 can also exert its effect on sodium channels via downstream signaling pathways in hippocampal neurons warrants future attention. Third, the inhibitory effect of anti‐PD‐1 treatment on neuronal excitability found by us was observed in the context of epilepsy‐related pathological changes. Previous studies have demonstrated that the expression pattern of sodium channels is altered during epileptogenesis, and the inactivation potential of sodium channels is depolarized in neurons after seizure.^44^, ^58^ Accordingly, it is reasonable that our study came to the opposite conclusion than others performed in DRG neurons, as the experimental models are different. Nonetheless, we only explored the effect of PD‐1 inhibition on neuronal excitability, and the effect of PD‐1 activation on neuronal excitability is also worth exploring.

However, the contradictory effects of PD‐1 blockade on hippocampal neurons are confusing. Effectively, the question is what are the differences between our experimental conditions and those of others? There is a key difference. We only tested the effects of PD‐1 blockade on epileptic hippocampal neurons but not normal neurons. Therefore, we repeated the experiment conducted by Zhao and Ji^55^ to test the effect of nivolumab on normal hippocampal CA1 neurons in acute brain slices. We were surprised to find that the excitability of hippocampal neurons in CA1 was lower in the nivolumab‐pretreated group (125.4 ± 16.3 pA, *n* = 13, *p* = 0.0048) than in the normal group (73.1 ± 7.8 pA, *n* = 16) (Figure S4B) as the injected current surpassed that required for firing of the first action potential but that the resting membrane potential was unaffected (Figure S4A). In addition, the number of action potentials was decreased after nivolumab incubation, as shown by the I‐O curves in Figure S4C, and typical traces of evoked action potentials in the two groups are shown in Figure S4D. The findings of this part of the experiment are highly consistent with our results regarding the effect of SHR‐1210 on epileptic hippocampal neurons and further confirm that anti‐PD‐1 treatment can inhibit the excitability of neurons.

However, there exist several notable limitations in the present study. One is the lack of brain samples from healthy subjects as a control, due to the inability to obtain such samples. In our present study, we investigated the potential role of PD‐1 in epilepsy by comparing its expression levels in the core lesion and the surrounding tissues and found that PD‐1 expression was significantly elevated in the core lesion. However, although the surrounding tissue has not yet exhibited spontaneous discharges, it may still be influenced by recurrent epileptic stimulation and undergo potential cellular changes. Therefore, including healthy samples as a control would be more convincing. The second is the absence of chronic epilepsy models such as pilocarpine‐ or kainic acid‐induced chronic temporal lobe epilepsy. The acute PTZ‐induced seizure model we employed offered valuable insights into the effects of anti‐PD‐1 treatment on immediate responses, and seizure susceptibility, however, was limited for understanding the long‐term effects of the PD‐1 monoclonal antibody on the progression of chronic epilepsy, the complexities of which include alterations in synaptic plasticity, network activity, and neuroinflammation.^59^ Therefore, future work to further investigate the impact of PD‐1 blockade on the progression and seizure onset of chronic epilepsy is valuable and needed. Last, while our intracellular recordings provide crucial information on the effects of SHR‐1210 on individual neuronal epileptiform activities, they fail to reflect changes in neuronal network activity, which always accompany epilepsy. Therefore, employing extracellular or microelectrode array (MEA) recordings to investigate the effects of SHR‐1210 on network excitability would be highly valuable and of great interest.

To summarize, our results support our hypothesis that the expression and function of PD‐1 is involved in epileptogenesis. Moreover, we found that the expression of PD‐1 is upregulated in the focal tissues responsible for seizure onset compared to perifocal tissues from human patients with intractable epilepsy. Given the role of PD‐1 in nociception,^15^, ^16^, ^17^ we revealed that PD‐1 might act as a modulator of sodium channels by regulating neuronal excitability through its interaction with Nav1.6. The anti‐epileptic effect of PD‐1 blockade is partly attributed to alteration of the electrophysiological properties of inactivated sodium channels. The present study provides evidence that PD‐1 participates in the generation of epilepsy, identifying PD‐1 as a novel endogenous epileptogenic factor and anti‐PD‐1 treatment as a novel therapeutic strategy for epilepsy and, potentially, for other neurological diseases.

### FUNDING INFORMARION

This work was funded by NSFC grants (81971204, 32111530119, 31771188) and supported by Shanghai Municipal Science and Technology Major Project (No. 2018SHZDZX01), ZJ Lab, and Shanghai Center for Brain Science and Brain‐Inspired Technology. It also partly supported by a grant from Shenzhen Science and Technology Innovation Commission Municipality (JCYJ20210324103409023) to LW and a grant from the Sanming Project of Medicine in Shenzhen (No. SZSM202111010) to YW.

## CONFLICT OF INTEREST STATEMENT

The authors declare no competing interests.

## PATIENT CONSENT

Informed consent was obtained from each patient for the use of brain tissue and access to medical records for research purposes.

## Supporting information


Figure S1.
Figure S2.
Figure S3.
Figure S4.
Table S1.

Data S1.

## Data Availability

All data generated or analyzed during this study are included in this published article (and its supplementary information files), or on request from the corresponding author.
