# Supplementary material for: Anti‐PD‐1 treatment protects against seizure by suppressing sodium channel function
Source: CNS Neurosci Ther. 2023 Oct 30;30(4):e14504. doi: 10.1111/cns.14504 (PMC11017438; doi:10.1111/cns.14504)
Supplement: Supplementary file 1 — Figure S1. Figure S2. Figure S3. Figure S4. Table S1. [file CNS-30-e14504-s001.docx]

Figure S1


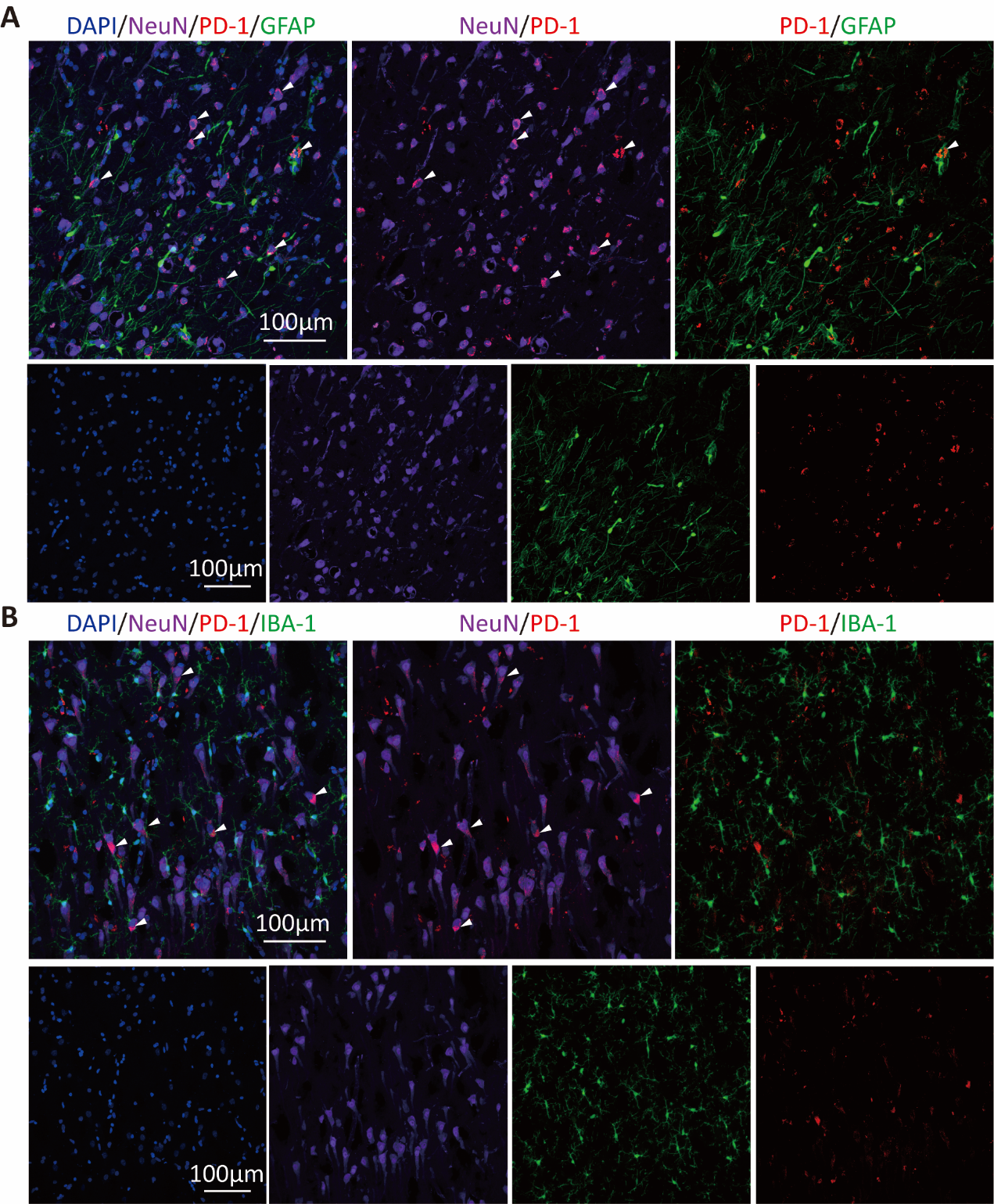


**Figure. S1. PD-1 is barely expressed in human astrocytes and microglia.**

(A) Immunostaining for PD-1, NeuN, and GFAP in focal tissue from patient #4. (B) Immunostaining for PD-1, NeuN and IBA-1 in focal tissue from patient #5. The white arrows point to neurons or microglia expressing PD-1.

Figure S2


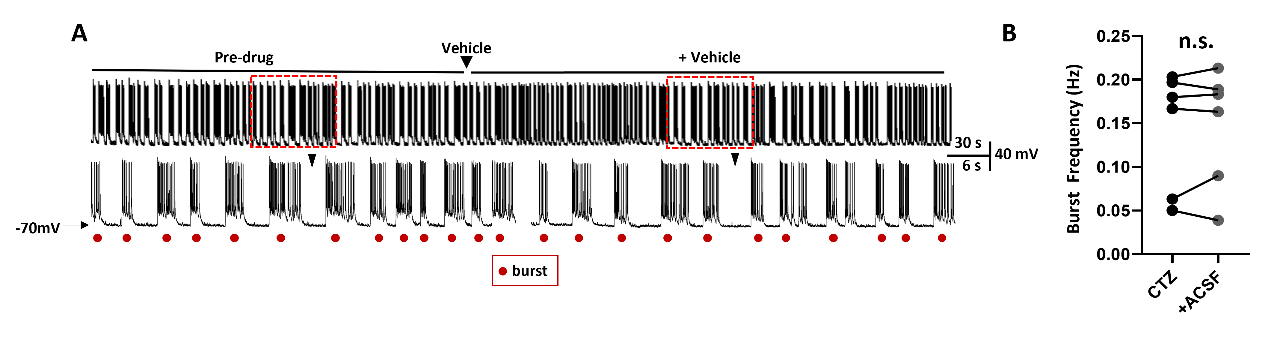


**Figure. S2. Continuous perfusion of ACSF containing no drug has no effect on neuronal excitability.**

(A) Representative neuronal activity and enlarged traces recorded before and after ACSF perfusion. (B) The burst frequency of hippocampal neurons before and after ACSF perfusion. (C) The percentage of burst discharges from hippocampal neurons before and after ACSF perfusion. The red dots indicate bursting activity induced by CTZ. Statistical analysis: Paired Student’s t test. (B, C). The data are the means ± SEMs.

Figure S3


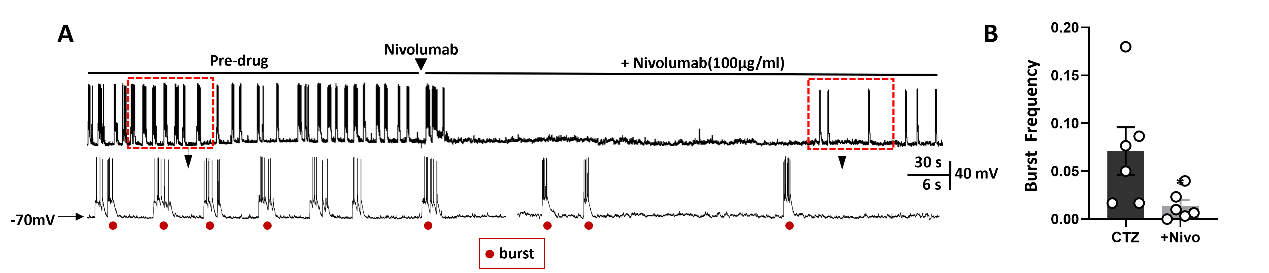


**Figure S3. Nivolumab can reduce the burst frequency of hippocampal neurons treated with CTZ.**

(A) Representative neuronal activity and enlarged traces recorded before and after perfusion with ACSF containing nivolumab. (B) The burst frequency of hippocampal neurons before and after perfusion with ACSF containing nivolumab. The red dots indicate bursting activity induced by CTZ. Statistical analysis: paired Student’s t test (B). The data are the means ± SEMs. *P < 0.05.

Figure S4


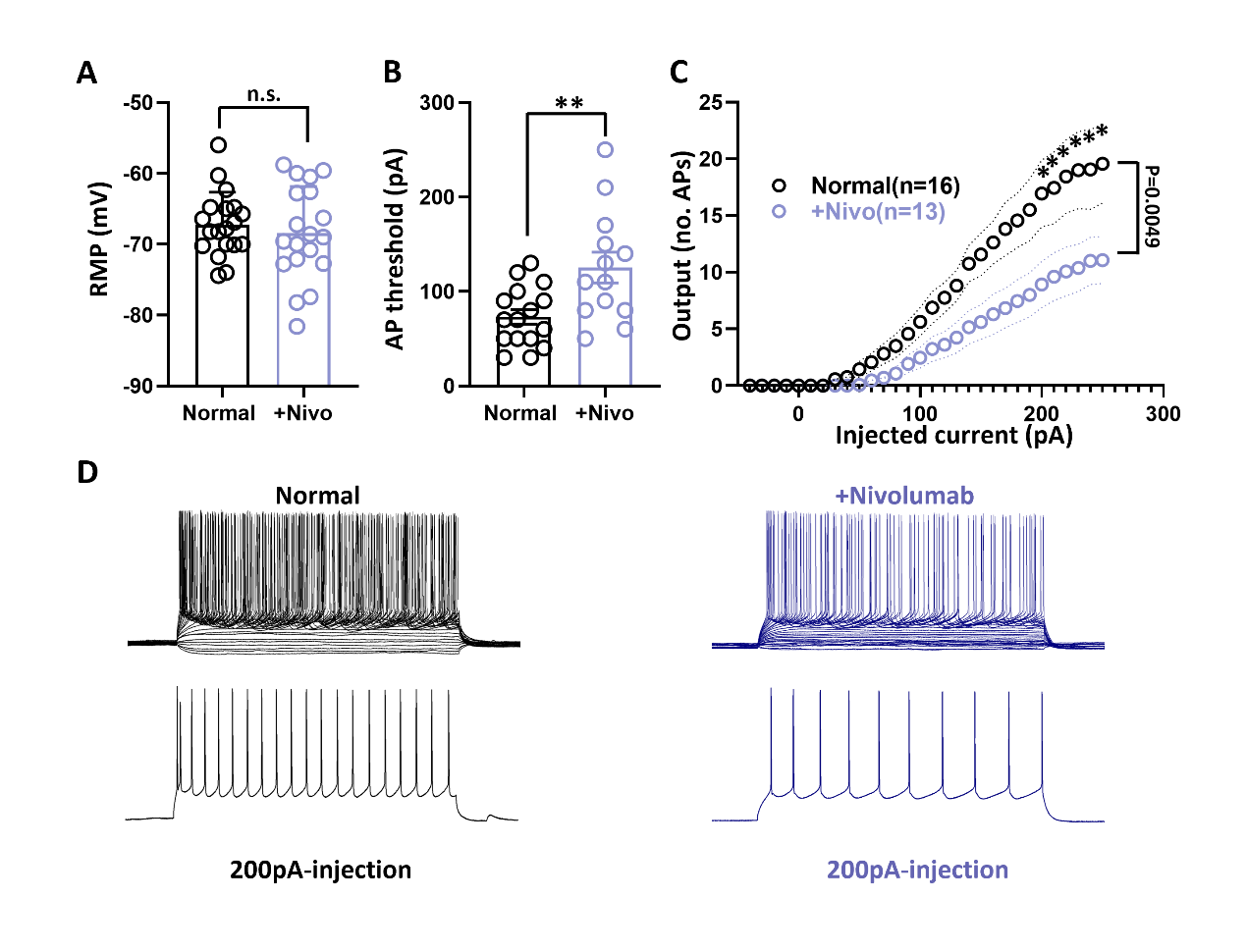


**Figure S4. The excitability of hippocampal neurons in the CA1 region was reduced after incubation with nivolumab.**

(A) The resting membrane potential (RMP) of hippocampal neurons in the CA1 region after incubation in normal ACSF and ACSF containing nivolumab (7 nM, 2 h). (B) Action potential threshold (the injected current needed to elicit the first action potential) of hippocampal neurons in the CA1 region. (C) Input‒output curves of hippocampal neurons treated with and without nivolumab. The dotted line indicates the SEM for each curve. (D) Representative action potentials of neurons incubated with and without nivolumab upon 200 pA current injection. Statistical analysis: unpaired Student’s t test (A, B) and two-way ANOVA (C). The data are the means ± SEMs. * P < 0.05, **P < 0.01.

Table S1 Patient information.

| **Patient (#)** | **1** | **2** | **3** | **4** | **5** | **6** | **7** | **8** | **9** |
| --- | --- | --- | --- | --- | --- | --- | --- | --- | --- |
| **Gender** | male | male | female | female | male | male | male | female | male |
| **Age at assessment** | 13 | 27 | 30 | 25 | 28 | 23 | 28 | 34 | 32 |
| **Seizure duration**  **(years)** | 11 | 10 | 23 | 11 | 7 | 5 | 10 | 18 | 20 |
| **ASMs before surgery** | VPA+PB+CBZ | OXC+TPM | PB+CBZ+TPM | LEV+LTG+VPA+CZP | OXC+VPA | VPA+TPM | TPM | OXC+LEV | VPA+TPM |
| **Region** | left parietal | left frontal | right parietal | right temporal | right frontal | left frontal | left temporoparietal | left temporal | right temporal |
| **Process** | PCR | PCR/WB | PCR/WB | PCR/WB/IF | PCR/WB/IF | WB | PCR/WB | PCR/WB | WB/IF |

VPA = Valproic acid; TPM = Topiramate; OXC = Oxcarbazepine; LEV = Levetiracetam; LTG = Lamotrigine; CZP = Clonazepam; PB = Phenobarbital; CBZ = Carbamazepine.
