# Supplementary figures and images for: Anti‐PD‐1 treatment protects against seizure by suppressing sodium channel function
Source: CNS Neurosci Ther. 2023 Oct 30;30(4):e14504. doi: 10.1111/cns.14504 (PMC11017438; doi:10.1111/cns.14504)

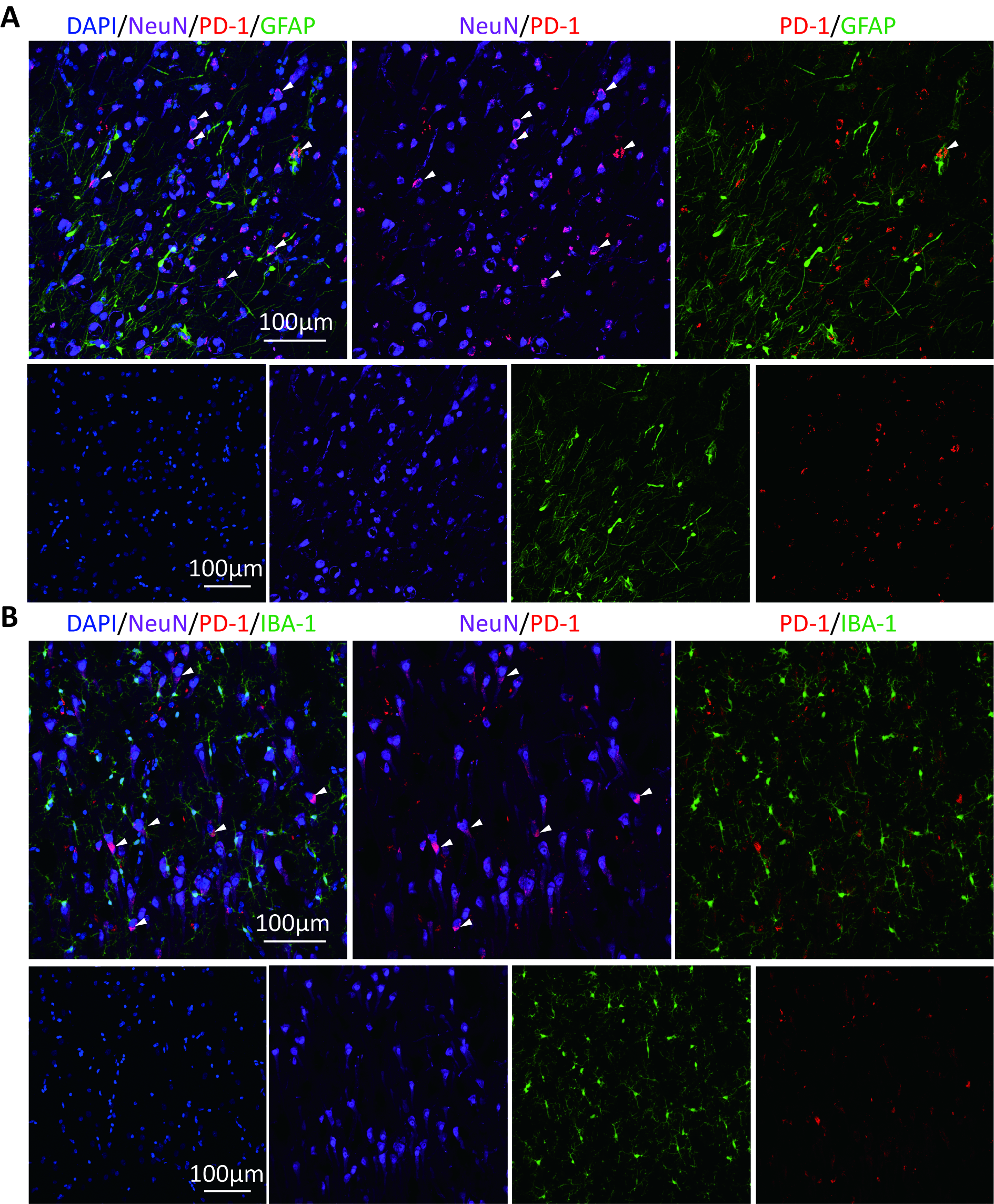

Supplement: Supplementary file 2 — Data S1. [file CNS-30-e14504-s002.zip › Fig S1.tif]

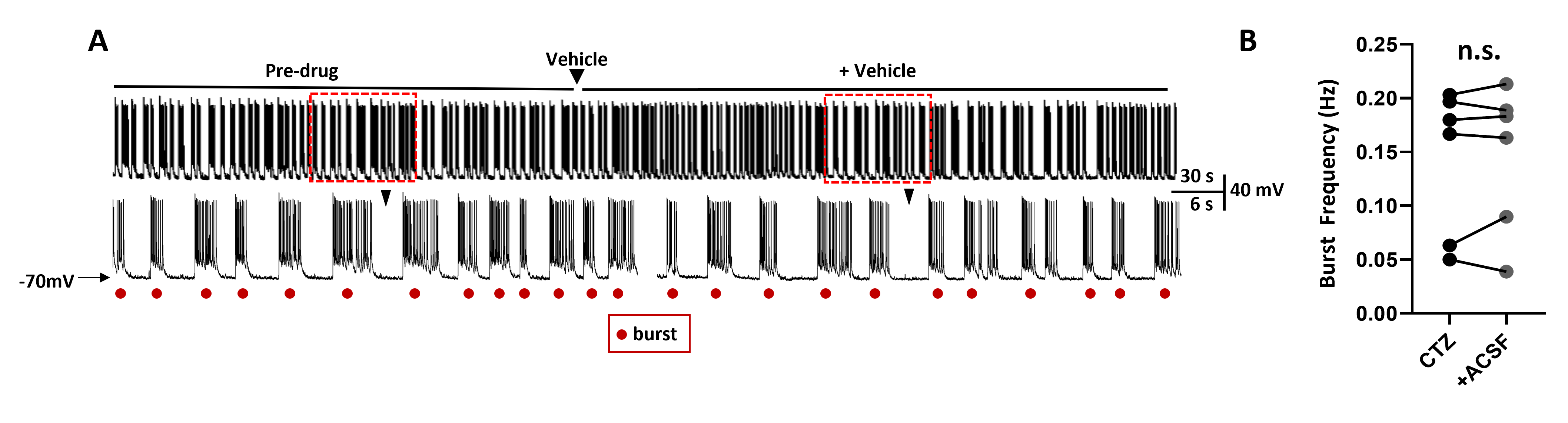

Supplement: Supplementary file 2 — Data S1. [file CNS-30-e14504-s002.zip › Fig S2.tif]

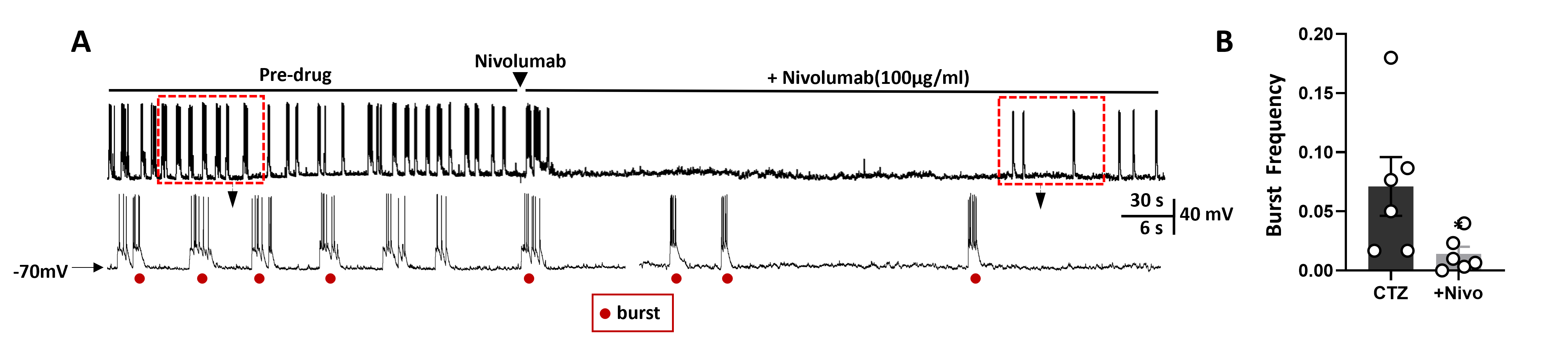

Supplement: Supplementary file 2 — Data S1. [file CNS-30-e14504-s002.zip › Fig S3.tif]

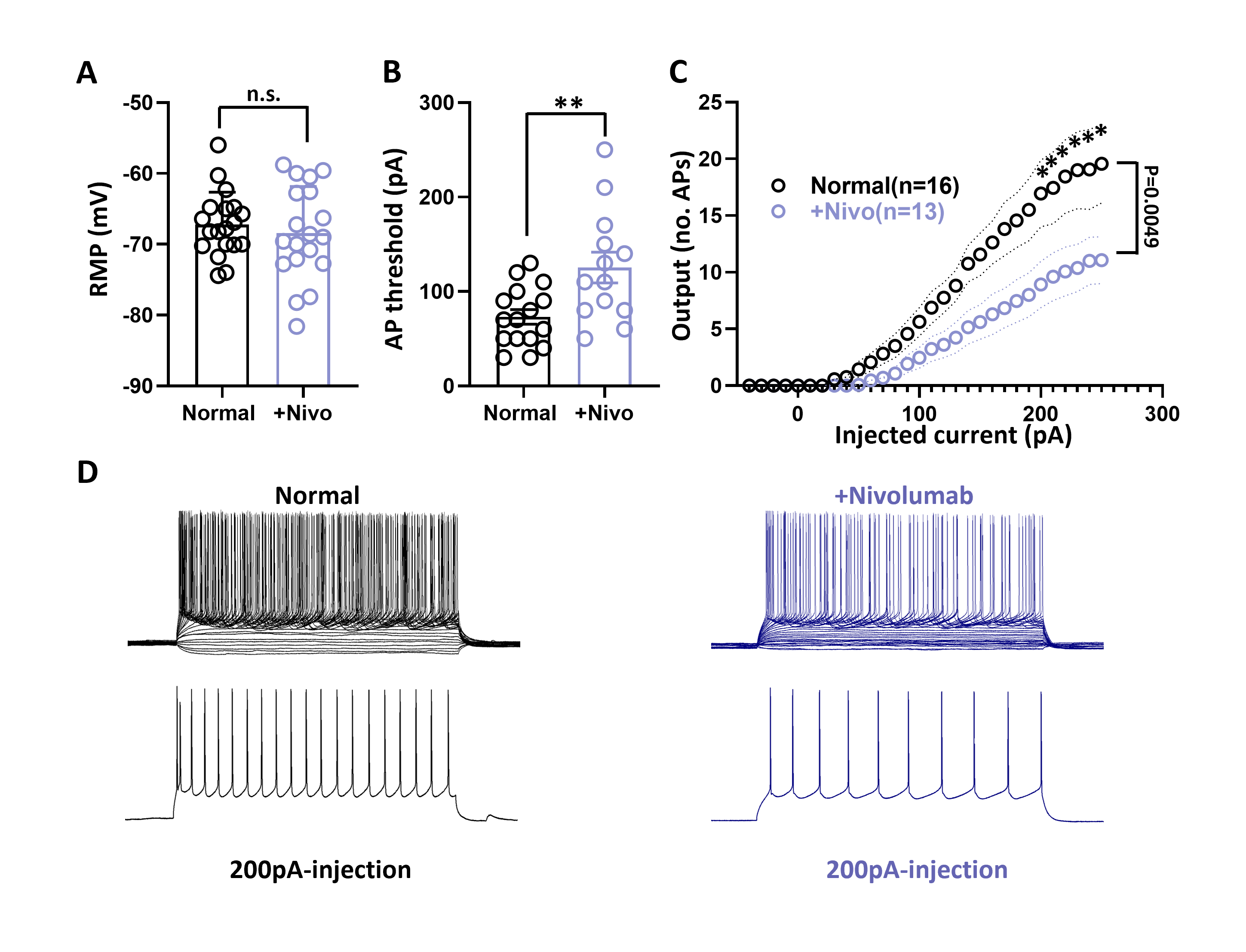

Supplement: Supplementary file 2 — Data S1. [file CNS-30-e14504-s002.zip › Fig S5.tif]

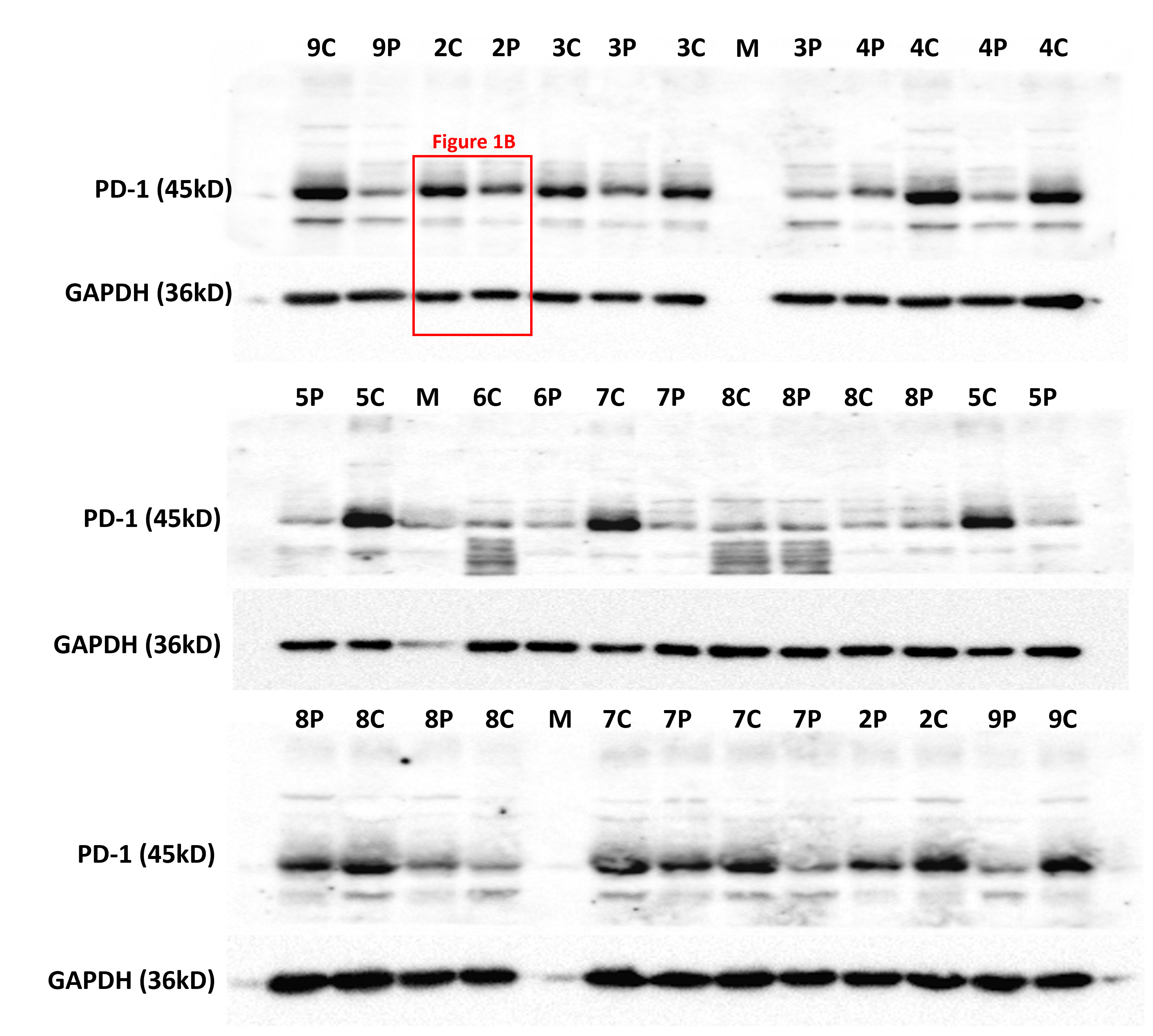

Supplement: Supplementary file 2 — Data S1. [file CNS-30-e14504-s002.zip › Figure S5.tif]

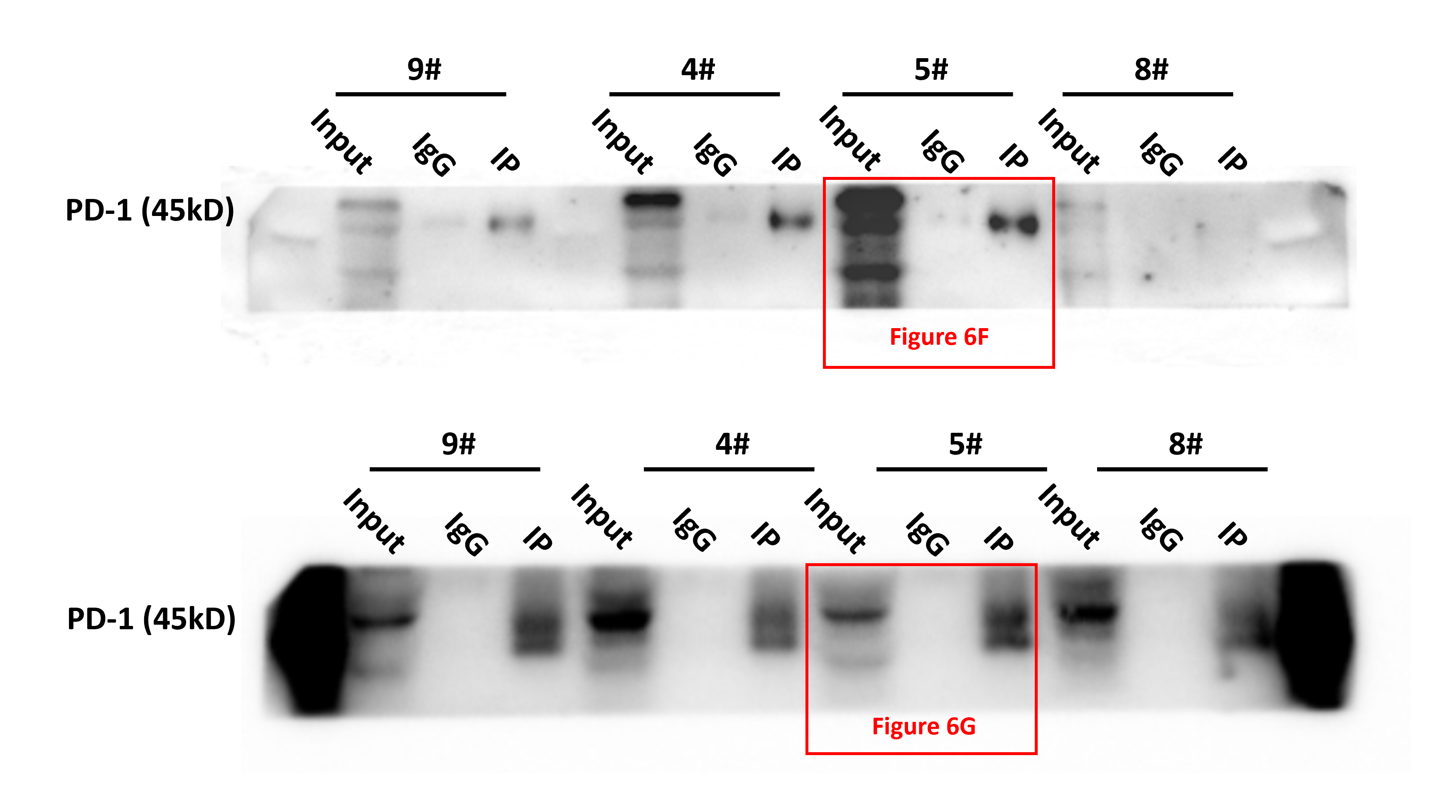

Supplement: Supplementary file 2 — Data S1. [file CNS-30-e14504-s002.zip › Figure S6.tif]
